# Development and validation of risk prediction model for premenstrual syndrome in nurses: results from the nurses-based the TARGET cohort study

**DOI:** 10.3389/fpubh.2023.1203280

**Published:** 2023-10-03

**Authors:** Li Li, Xiaoyan Lv, Yuxin Li, Xinyue Zhang, Mengli Li, Yingjuan Cao

**Affiliations:** ^1^Department of Nursing, Qilu Hospital, Cheeloo College of Medicine, Shandong University, Jinan, China; ^2^Nursing Theory and Practice Innovation Research Center, Cheeloo College of Medicine, Shandong University, Jinan, Shandong, China; ^3^Department of Neonatology, Shandong Provincial Hospital, Jinan, China; ^4^School of Nursing and Rehabilitation, Cheeloo College of Medicine, Shandong University, Jinan, China

**Keywords:** premenstrual syndrome, nurses, risk factors, nomogram, model validation

## Abstract

**Objective:**

Premenstrual syndrome (PMS) stands as a significant concern within the realm gynecological disorders, profoundly impacting women of childbearing age in China. However, the elusive nature of its risk factors necessitates investigation. This study, therefore, is dedicated to unraveling the intricacies of PMS by focusing on nurses, a cohort with unique occupational stressors, to develop and validate a predictive model for assessing the risk of PMS.

**Methods:**

This investigation employed a multi-center cross-sectional analysis drawing upon data from the TARGET Nurses’ health cohort. Utilizing online survey versions of the Premenstrual Syndrome Scale (PMSS), a comprehensive dataset encompassing physiological, social, psychological, occupational, and behavioral variables was collected from 18,645 participants. A stepwise multivariate logistic regression analysis was conducted to identify independent risk factors for PMS. Furthermore, a refined variable selection process was executed, combining the Least Absolute Shrinkage and Selection Operator (LASSO) method with 10-fold cross-validation. The visualization of the risk prediction model was achieved through a nomogram, and its performance was evaluated using the C index, receiver operating characteristic (ROC) curves, and the calibration curves.

**Results:**

Among the diverse variables explored, this study identified several noteworthy predictors of PMS in nurses, including tea or coffee consumption, sleep quality, menstrual cycle regularity, intermenstrual bleeding episodes, dysmenorrhea severity, experiences of workplace bullying, trait coping style, anxiety, depression and perceived stress levels. The prediction model exhibited robust discriminatory power, with an area under the curve of 0.765 for the training set and 0.769 for the test set. Furthermore, the calibration curve underscored the model’s high degree of alignment with observed outcomes.

**Conclusion:**

The developed model showcases exceptional accuracy in identifying nurses at risk of PMS. This early alert system holds potential to significantly enhance nurses’ well-being and underscore the importance of professional support.

## 1. Introduction

Premenstrual syndrome (PMS) constitutes a constellation of emotional, behavioral, and physical symptoms that typically emerge during the luteal phase of the female menstrual cycle, manifesting 7–14 days before menstruation and subsequently abating. These symptoms encompass emotional manifestations such as irritability, anxiety, and depression, behavioral symptoms like sleep disorders and reduced attention, as well as physical complaints like headaches and diarrhea (1). Traditionally acknowledged as a concern among women of childbearing age (2), PMS is fast becoming a common mental disease, substantially impairing the ability of quite a few women to daily function. Meanwhile, increased incidence of some symptoms on PMS in the context of the COVID-19 pandemic (3). An examination of gynecological disease burden data in China underscores PMS as one of the top three gynecological diseases affecting women aged 15 to 49 (4, 5).

A comprehensive review of literature spanning the past five years reveals that PMS affects 25 to 70% of women across all age groups (2, 6–8). This is a matter of profound concern, given the substantial impact of PMS on both physical and mental well-being, as well as its disruptive influence on daily life and work (9). Moreover, PMS significantly increases suicidal ideation in patients, which is a significant risk factor for depression, postpartum depression, frequent work absenteeism and reduced work productivity (10–12). Therefore, the evaluation and effective management of PMS constitute crucial clinical imperatives, given its far-reaching implications.

Remarkably, prior investigations into the prevalence and associated factors of PMS have primarily focused on adult students (3, 13–15), with relatively limited attention directed toward specific occupational groups, including nurses. Clinical nurses, as the custodians of patient well-being, play pivotal roles in healthcare delivery. Owing to the demanding nature of their work and the associated pressures, nurses may be particularly susceptible to PMS, which can reduce their quality of life (16). It is important to note that PMS may be influenced by a variety of factors, encompassing personal characteristics, lifestyle habits, occupational attributes, physiological variables, psychological variables and social determinants. Several studies have linked factors such as underweight, obesity (17, 18), smoking (19), and consumption of coffee and tea (20) to an increased risk of PMS. Additionally, factors inherent to nursing professions, including shift work and sleep patterns, have been identified as risk factors for PMS (21, 22). Physiological variables, such as the intensity of menstrual pain, irregular menstrual cycles, early menarche, prolonged menstruation, and the use of excessive menstrual pads, have also shown significant associations with PMS (15). Other factors impacting PMS include physical activity (23), stress levels (18), personality traits (24), burnout and social support (25). However, PMS often goes unnoticed due to its absence of a clear developmental process. Given the multitude of related risk factors, accurate prediction and early intervention are effective strategies for reducing the incidence of PMS.

In summary, there exists a notable gap in research pertaining to the development of predictive models for assessing PMS risk in nurses, and lack of risk assessment tools for early individualized assessment by nurses themselves. Our study endeavors to fill this void by establishing a nomogram capable of predicting the risk factors for PMS among nurses. Which can early recognize of PMS and could mostly benefit nurses with targeted risk factors. This tool seeks to enable the early recognition of PMS and offer targeted support to nurses with identifiable risk factors. Through an exhaustive analysis and integration of risk factors that exert influence over the onset, development, and prognosis of PMS, our research aims to empower head nurses and nurses to identify PMS early and prompt them to actively seek professional assistance.

## 2. Materials and methods

### 2.1. Study population

**Figure 1:**
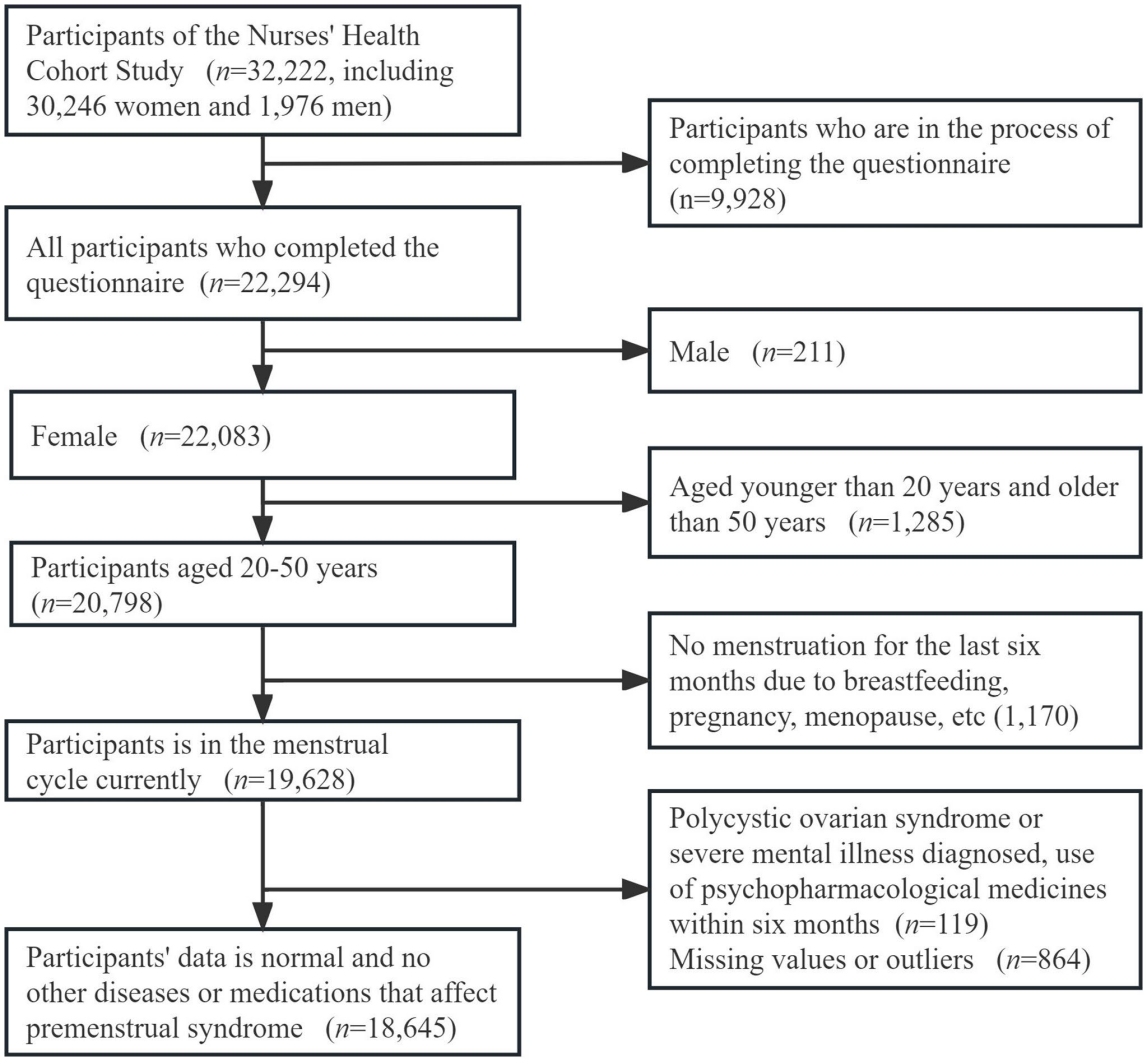
Flow chart of inclusion and exclusion for the final study population in this study.

The TARGET Nurses’ health cohort study (26), launched in December 2020, is a prospective cohort study that focuses on nurses in China. For our study, we harnessed data from the first survey, which recruited participants aged 18–65 with nurse qualification certificates. Participants across Shandong Province were recruited from 46 public hospitals of different grades. In total, 32,222 participants agreed to take part in the study until November 2022. Among these participants, 22,924 were dedicated nurses who diligently completed the comprehensive questionnaire. This nurse cohort was composed of 22,083 females and 211 males. To refine our study cohort further, we meticulously applied exclusion criteria to the subset of 20,798 women aged between 20 and 50 years. Exclusion criteria are set below: (a) nurses who had not experienced menstruation for the past six months due to breastfeeding, pregnancy, menopause, etc. (*n* = 1,170); (b) those with a prior diagnosis of polycystic ovarian syndrome, or severe mental illness, or those who had used psychopharmacological medicines within six months preceding the completion of the women’s questionnaire (*n* = 119). Additionally, nurses whose questionnaires exhibited missing data values or outliers across all variables were also excluded from the final analytical dataset (*n* = 864) (Figure 1).

All participants provided their informed consent prior to their involvement in the study. The study protocol secured approval from the Ethics Committee and was duly registered in the clinical trial registry.

### 2.2. Measures

The dataset employed in this study encompassed four distinct sections, comprising one dependent variable and 28 independent variables. These variables included the following categories: general information of participants (age, highest educational level, monthly income, marital status, and body mass index), physiology factors (menarcheal age, length of the menstrual cycle, regularity of the menstrual cycle, menstrual period, menstrual blood flow, number of intermenstrual bleedings, the degree of dysmenorrhea, and number of pregnancies), occupational and behavioral factors (nap frequency, smoking, drinking, milk, tea or coffee, physical activity, sleep quality, shifts, and work experience), psychological and social factors, were measured as described below (trait coping style, experiences of workplace bullying, social support, stress, anxiety, and depression).

#### 2.2.1. General information of participants

This study collected data encompassing key demographic attributes of participants, including age, highest educational level achieved, monthly income, marital status, and body mass index, which were classified as underweight (< 18.5 kg/m^2^), normal weight (18.5–23.9 kg/m^2^), and overweight (24–27.9 kg/m^2^), obesity (> 27.9 g/m^2^), were collected from each participant.

#### 2.2.2. Premenstrual syndrome scale

To assess the severity of PMS symptoms, we harnessed the widely employed Premenstrual Symptoms Scale (PMSS), which is renowned for its simplicity, expeditiousness, and effectiveness. In this study, we adopted the Chinese version (27) of PMSS (28) to measure the extent of PMS symptoms experienced by participants. The scale has 12 items, encompassing psychological symptoms (irritability, depression, anxiety, inattention, nervousness, fidgeting, neuroticism) and physical symptoms (bloating and diarrhea, sleepiness, migraine, insomnia, swelling of hands and feet). Participants were required to indicate whether these symptoms manifested within 14 days before the last menstrual period. Each symptom was graded on a scale of 0–3, with no symptoms (0); mild symptoms (1); deemed tolerable while affecting daily life and work (2), and symptoms seriously impeding daily life and work, necessitating treatment (3). The scale is calculated as the sum of the scores of each item (range 0–36 points). In our study, individuals scoring above 5 were categorized as symptomatic, whereas those scoring below 5 were considered asymptomatic. The Cronbach’s alpha coefficient for the scale was 0.930 in this study.

#### 2.2.3. Physiology factors

Participants were prompted to provide information pertaining to menstrual characteristics and any alterations observed in the past six months, including menarcheal age (< 12 years, 12–14 years, 15–17 years, > 17 years), length of the menstrual cycle (< 25 days, 26–31 days, 32–39 days, > 39 days), regularity of the menstrual cycle, defined by the number of days between two menstrual cycles (within 1–2 days, within 3–4 days, within 5–7 days, more than 7 days, irregular); menstrual period (3–7 days, < 3 days, > 7 days, irregular), menstrual blood flow, quantified by the number of sanitary napkins used during each menstrual period (less than 1 pack of sanitary napkins, 1 or 2 packs of sanitary napkins, more than 2 packs of sanitary napkins), number of intermenstrual bleedings (< 1 time, 1–3 times, > 3 times) and the degree of dysmenorrhea (none, mild, moderate, severe). Additionally, reproductive factors included the number of pregnancies, categorized as 0, 1, 2, and ≥ 3 times.

#### 2.2.4. Occupational and behavioral factors

In the realm of occupational considerations, our study delved into the nurses’ work patterns and their cumulative work experience. To further delineate their behavioral attributes, we employed the following inquiries: (a) How many naps do you take each week? (b) How often do you smoke or drink? And (c) How often do you consume milk, tea or coffee?

##### 2.2.4.1. International physical activity questionnaire

For the assessment of physical activity levels, we harnessed the International Physical Activity Questionnaire (IPAQ). Originating from the World Health Organization in 1998 (29), this instrument has been adeptly adapted into the Chinese context by Qu Ningning in 2004 and has found widespread utility (30). The IPAQ prompts participants to recall their physical activities over the preceding week, including 4 types of physical activities: occupational, household, transportation, and leisure, with 24 entries. For each item denoting a specific physical activity, participants meticulously recorded the duration of engagement. Finally, the types and durations of activities were converted into metabolic equivalents (MET) to measure physical activity levels, classified as low, medium, or high.

##### 2.2.4.2. The Pittsburgh sleep quality index

The Pittsburgh Sleep Quality Index (PSQI) scale was provided to all participants to assess their sleep quality (31). We used the Chinese scale to evaluate nurses’ sleep quality and time. The scale has been used widely and has good reliability and validity (32). The scale consists of 18 self-evaluation items and five other-evaluation items. The latter part is not scored. The former part is composed of seven dimensions, including sleep quality, sleep time, sleep efficiency, sleep disorder, hypnotic drugs and daytime dysfunction factors. The total score ranges from 0 to 21. The higher the score, the worse the sleep quality. In this study, a PSQI score of <6 was indicative of favorable sleep quality, and a score of≥6 indicated a deterioration in sleep quality. The Cronbach’s alpha coefficient for the scale was 0.798 in this study.

#### 2.2.5. Psychological and social factors

##### 2.2.5.1. Negative acts questionnaire

The Negative Acts Questionnaire-Revised (NAQ-R) (33) was used to measure the workplace bullying directed toward nurses by their leaders and colleagues. The Chinese version of the NAQ-R, widely adopted (34), encompasses three dimensions featuring 23 items, including nine items for personal-related negative behaviors, nine items for work-related negative behaviors, and four items for organizational injustice. The initial 22 items were scored on a 5-point Likert scale, ranging from 1 (never) to 5 (every day), with the 23^rd^ item prompting participants to self-assess the level of workplace bullying that they had experienced. Based on the total score, exposure to bullying was classified into three categories: no bullying (< 33), frequent bullying (33–45), and exposure to severe bullying exposure (> 45). The Cronbach’s alpha coefficient for the scale was 0.978 in this study.

##### 2.2.5.2. Perceived social support scale

The assessment of social support levels drew upon the Perceived Social Support Scale (PSSS) (35). The Chinese version (36) of the PSSS used in this study has been proven reliable. It has 12 items and is divided into three dimensions: support from family, friends, and others, providing insights into support both within and beyond the family context. Responses for each item range from 1 to 7, with higher scores denoting elevated levels of social support. Categorized into three tiers, the scale delineates low support (12–36 points), intermediate support (37–60 points), and relatively high support (61–84 points). Widely employed in China, the Cronbach’s alpha coefficient for the scale was 0.989 in this study.

##### 2.2.5.3. Trait coping style questionnaire

To delve into personality traits and their reflection in coping strategies, we deployed the Trait Coping Style Questionnaire (TCSQ) (37). The questionnaire consists of 20 items, partitioned into two dimensions: positive coping and negative coping, each of which contains 10 items. Employing a 5-point Likert scoring method (1–5), the TCSQ ascertains respondents’ tendencies toward positive or negative attitudes and behavioral characteristics when confronting stressors. Higher scores within each dimension correspond to more pronounced positive or negative coping strategies. The Cronbach’s alpha coefficient for the scale was 0.907 in this study.

##### 2.2.5.4. Perceived stress scale

To gauge the level of perceived stress among participants in their daily lives over the past month, we employed the Perceived Stress Scale (PSS), grounded in psychological stress theory and developed in 1983 (38). Introduced into China in 2003 (39), this scale boasts wide utilization and robust reliability and validity. The scale consists of 10 items on a five-point scale of 0–4, representing “never,” “occasionally,” “sometimes,” “often,” and “always” respectively. The total score ranges from 0 to 40. In this study, the level of perceived stress was classified as low stress (<14), medium stress (14–26), and high stress (>26). The Cronbach’s alpha coefficient for the scale in this study was 0.934.

##### 2.2.5.5. Anxiety severity

The Generalized Anxiety Disorder 7-Item Scale (GAD-7) (40) developed by Spitzer et al. has been widely used to screen for anxiety and assess its severity. The reliability and validity of the Chinese version of the GAD-7 have been established by He et al. who found the test–retest reliability to be 0.830 and a Cronbach’s α of 0.920 indicating good reliability and validity (41). The total score ranges from 0 to 21, with each of the seven items scoring from zero (not at all) to three (almost every day), so that scores of 0–4, 5–9, 10–14, and 15–21 represent normal, mild, moderate, and severe anxiety, respectively. The Cronbach’s α of the GAD-7 was 0.963 in this study.

##### 2.2.5.6. Depressed severity

The 9-item Patient Health Questionnaire (PHQ-9) was developed by Kroenke et al. and has been widely used to assess depressive symptoms (42). The PHQ-9 was translated into Chinese by Wang et al., has demonstrated sound psychometric properties, boasting a test–retest reliability of 0.860 and a Cronbach’s alpha of 0.860 (43). The total score ranges from 0 to 27 points, with each of the nine items scoring from zero (not at all) to three (nearly every day), so scores of 0–4, 5–9, 10–14, 15–19, and 20–27 represent normal, mild, moderate, moderately severe, and severe depression, respectively. The Cronbach’s α of the PHQ-9 was 0.955 in this study.

### 2.3. Data collection and quality control

The data for this study were collected from the baseline data of TARGET, accessible through electronic questionnaires made available on the official TARGET WeChat platform (26). These questionnaires have been validated in Chinese. All participants granted electronic informed consent via the WeChat platform, affirming their independent and anonymous completion of the questionnaire. This informed consent emphasized the voluntary nature of participation, affirming the unequivocal right of individuals to opt out if they so choose.

To ensure the quality and validity of the data, we adopted a comprehensive approach to quality control. A second reminder was given to all non-participants via WeChat one week after the initial questionnaire distribution, with a subsequent reminder issued after an equivalent interval. The electronic questionnaire was pre-set to enforce the completion of mandatory questions and integrate skip logic for certain items. This careful questionnaire formatting ensured the standardization and completeness of responses. Additionally, participants were allocated a specific response time window, ranging from 15 to 20 min, further enhancing data quality. Liaison officers at each participating hospital supervised the completion of the questionnaires, facilitating communication with researchers in cases where issues required resolution beyond routine channels.

### 2.4. Statistical analysis

All analyses were conducted using R software version 4.0.3 (R Development Core Team, Vienna, Austria). This study included one dependent variable and 28 independent variables in this study, all of which classified variables, expressed as frequencies and percentages. The characteristic distribution between nurses with PMS and non-PMS was evaluated by chi-square test or Fisher’s exact classification variable test. The analytical course followed this trajectory: First, the Least Absolute Shrinkage and Selection Operator (LASSO) method was used to sift through the variables of interest, pinpointing pivotal variables by the glmnet and caret packages. Subsequently, a multivariate logistic regression analysis was embarked upon for the selected variables, streamlining the variables and culminating in the construction of the risk prediction model. The visual representation of this model materialized through a nomogram, skillfully generated via the rms and regplot packages, with the principal aim of providing a readily interpretable snapshot of the predictive model. Decision curve analysis and ROC curves determined sensitivity and specificity, while the discrimination and calibration of the model were subject to scrutiny via ROC and calibration curves, fashioned with the assistance of the pROC and ggplot2 packages. The comprehensive validation spanned internal and external aspects, leveraging the power of 1,000 Bootstrap self-sampling iterations. Statistical significance hinged on a two-tailed *p* value threshold of less than 0.05.

## 3. Results

### 3.1. Sample and demographic characteristics

A total of 18,645 participants were included in this study for analysis (Figure 1). Among these participants, 8,216 were nurses diagnosed with PMS, while 10,429 were without PMS. The participants had a mean age of 33.50 years with a standard deviation of 5.89 years and 10.65 ± 6.40 for working years. The average BMI was 22.30 ± 3.11 kg/m^2^, and about 76.6% of the participants were married. Table 1 summarizes the clinical characteristics, encompassing physiological, occupational and behavioral, psychological, and social factors for the participants with PMS and non-PMS. In addition, for model validation, the data were divided into a training set and a test set in a 7: 3 ratio. Table 2 outlines the clinical characteristics of the participants in both sets. There were no significant differences among these characteristics, indicating the absence of bias arising from random grouping.

**Table 1:** The Clinical characteristics of nurses with the PMS and non-PMS.

Variables	PMS *n* (%) (*n* = 10,429)	Non-PMS *n* (%) (*n* = 8,216)	*p*-value
Age			< 0.001^**^
20–25 years	3,781 (36.3%)	3,048 (37.1%)	
26–30 years	993 (9.5%)	846 (10.3%)	
31–35 years	1,995 (19.1%)	1,822 (22.2%)	
36–40 years	2,156 (20.7%)	1,682 (20.5%)	
41–45 years	1,010 (9.7%)	595 (7.2%)	
46–50 years	494 (4.7%)	223 (2.7%)	
Education level			0.098
College degree or below	787 (7.5%)	568 (6.9%)	
Undergraduate degree or above	9,642 (92.5%)	7,648 (93.1%)	
Marital status			< 0.001^**^
Unmarried	2,143 (20.5%)	1,925 (23.4%)	
Married	8,133 (78.0%)	6,146 (74.8%)	
Others	153 (1.5%)	145 (1.8%)	
Work experience			< 0.001^**^
0–5 years	2,824 (27.1%)	2,396 (29.2%)	
6–10 years	3,249 (31.2%)	2,681 (32.6%)	
11–15 years	2,422 (23.2%)	1,895 (23.1%)	
> 15 years	1,934 (18.5%)	1,244 (15.1%)	
Monthly income			0.113
< 3,000 yuan	831 (8.0%)	699 (8.5%)	
3,000–6,000 yuan	5,498 (52.7%)	4,381 (53.3%)	
6,000–9,000 yuan	3,120 (29.9%)	2,436 (29.6%)	
> 9,000 yuan	980 (9.4%)	700 (8.5%)	
Body mass index			0.266
< 18.5 kg/m^2^	6,622 (63.5%)	5,160 (62.8%)	
18.5–23.9 kg/m^2^	966 (9.3%)	814 (9.9%)	
24–27.9 kg/m^2^	2,349 (22.5%)	1,823 (22.2%)	
> 27.9 g/m2	492 (4.7%)	419 (5.1%)	
Smoking			0.409
Non-smoker	10,424 (100.0%)	8,214 (100.0%)	
Smoker	5 (0.0%)	2 (0.0%)	
Drinking			0.341
Non-drinker	10,407 (99.8%)	8,193 (99.7%)	
Drinker	22 (0.2%)	23 (0.3%)	
Tea or coffee			< 0.001^**^
No	7,451 (71.4%)	5,292 (64.4%)	
Yes	2,978 (28.6%)	2,924 (35.6%)	
Milk			< 0.001^**^
No	3,792 (36.4%)	2,794 (34.0%)	
Yes	6,637 (63.6%)	5,422 (66.0%)	
Menarcheal age			0.160
< 12 years	7,972 (76.4%)	6,186 (75.3%)	
12–17 years	2,254 (21.6%)	1,872 (22.8%)	
> 17 years	203 (1.9%)	158 (1.9%)	
Length of the menstrual cycle			< 0.001^**^
< 26 days	235 (2.3%)	235 (2.9%)	
26–31 days	8,330 (79.9%)	6,232 (75.9%)	
32–39 days	1,357 (13.0%)	1,191 (14.5%)	
> 39 days	507 (4.9%)	558 (6.8%)	
Regularity of the menstrual cycle			< 0.001^**^
Within 1–2 days	2,091 (20.0%)	1,119 (13.6%)	
Within 3–4 days	3,870 (37.1%)	2,597 (31.6%)	
Within 5–7 days	2,348 (22.5%)	2,106 (25.6%)	
> 7 days	1,313 (12.6%)	1,474 (17.9%)	
Irregular	807 (7.7%)	920 (11.2%)	
Menstrual period			< 0.001^**^
< 3 days	492 (4.7%)	473 (5.8%)	
3–7 days	9,016 (86.5%)	6,689 (81.4%)	
> 7 days	802 (7.7%)	862 (10.5%)	
Irregular	119 (1.1%)	192 (2.3%)	
Menstrual blood flow^#^			< 0.001^**^
Less than 1 pack	1,534 (14.7%)	1,148 (14.0%)	
1 or 2 packs	6,156 (59.0%)	4,595 (55.9%)	
More than 2 packs	2,739 (26.3%)	2,473 (30.1%)	
Number of bleeding between menstruation			< 0.001^**^
< 1 times	9,043 (86.7%)	6,451 (78.5%)	
1–3 times	1,228 (11.8%)	1,498 (18.2%)	
> 3 times	158 (1.5%)	267 (3.2%)	
The degree of dysmenorrhea			< 0.001^**^
No	2,829 (27.1%)	1,055 (12.8%)	
Melt	5,505 (52.8%)	3,587 (43.7%)	
Moderate	1,650 (15.8%)	2,692 (32.8%)	
Severe	445 (4.3%)	882 (10.7%)	
Number of pregnancies			< 0.001^**^
0	2,862 (27.4%)	2,559 (31.1%)	
1	2,486 (23.8%)	1,897 (23.1%)	
2	2,774 (26.6%)	1,946 (23.7%)	
> = 3	2,307 (22.1%)	1,814 (22.1%)	
Shift pattern			< 0.001^**^
Only day shift	3,492 (33.5%)	2,313 (28.2%)	
Two shifts	3,533 (33.9%)	2,961 (36.0%)	
Three shifts	3,267 (31.3%)	2,779 (33.8%)	
Others	137 (1.3%)	163 (2.0%)	
Nap per week			< 0.001^**^
1–2	3,064 (29.4%)	2,641 (32.1%)	
3–4	3,005 (28.8%)	2,634 (32.1%)	
> = 5	4,360 (41.8%)	2,941 (35.8%)	
Sleep quality			< 0.001^**^
Well	7,568 (72.6%)	3,936 (47.9%)	
Poor	2,861 (27.4%)	4,280 (52.1%)	
Level of bullying			< 0.001^**^
No	9,235 (88.6%)	5,861 (71.3%)	
Often	804 (7.7%)	1,474 (17.9%)	
Always	390 (3.7%)	881 (10.7%)	
Level of social support			< 0.001^**^
Low	1,928 (18.5%)	1,364 (16.6%)	
Medium	2,880 (27.6%)	3,252 (39.6%)	
High	5,621 (53.9%)	3,600 (43.8%)	
Trait coping style			< 0.001^**^
Positive	6,768 (64.9%)	3,931 (47.8%)	
Neutral	2,371 (22.7%)	2,493 (30.3%)	
Negative	1,290 (12.4%)	1,792 (21.8%)	
Anxiety			< 0.001^**^
No	6,111 (58.6%)	2,105 (25.6%)	
Melt	3,518 (33.7%)	4,188 (51.0%)	
Moderate	562 (5.4%)	1,268 (15.4%)	
Severe	238 (2.3%)	655 (8.0%)	
Depression			< 0.001^**^
No	5,885 (56.4%)	1,839 (22.4%)	
Melt	3,547 (34.0%)	3,945 (48.0%)	
Moderate	514 (4.9%)	1,035 (12.6%)	
Severe	325 (3.1%)	943 (11.5%)	
Extremely severe	158 (1.5%)	454 (5.5%)	
Physical activity			< 0.001^**^
Low	3,365 (32.3%)	2,556 (31.1%)	
Medium	2,381 (22.8%)	1,728 (21.0%)	
High	4,683 (44.9%)	3,932 (47.9%)	
Perceived stress			< 0.001^**^
Low	2,382 (22.8%)	802 (9.8%)	
Medium	7,911 (75.9%)	6,963 (84.7%)	
High	136 (1.3%)	451 (5.5%)	

** Significant at *p* < 0.001.

# Menstrual volume was estimated by the number of sanitary napkins used (one packet is 10 pieces).

**Table 2:** The clinical characteristics of nurses in the training and test set.

Variables	Training set *n* (%) (*n* = 13,053)	Test set *n*(%) (*n* = 5,592)	*p*-value
Age			0.489
20–25 years	4,808 (36.8%)	2,021 (36.1%)	
26–30 years	1,300 (10.0%)	539 (9.6%)	
31–35 years	2,656 (20.3%)	1,161 (20.8%)	
36–40 years	2,645 (20.3%)	1,193 (21.3%)	
41–45 years	1,142 (8.7%)	463 (8.3%)	
46–50 years	502 (3.8%)	215 (3.8%)	
Education level			0.475
College degree or below	937 (7.2%)	418 (7.5%)	
Undergraduate degree or above	12,116 (92.8%)	5,174 (92.5%)	
Marital status			0.305
Unmarried	2,809 (21.5%)	1,259 (22.5%)	
Married	10,032 (76.9%)	4,247 (75.9%)	
Others	212 (1.6%)	86 (1.5%)	
Work experience			0.697
0–5 years	3,629 (27.8%)	1,591 (28.5%)	
6–10 years	4,167 (31.9%)	1,763 (31.5%)	
11–15 years	3,043 (23.3%)	1,274 (22.8%)	
>15 years	2,214 (17.0%)	964 (17.2%)	
Monthly income			0.214
<3,000 yuan	1,052 (8.1%)	478 (8.5%)	
3,000–6,000 yuan	6,942 (53.2%)	2,937 (52.5%)	
6,000–9,000 yuan	3,856 (29.5%)	1,700 (30.4%)	
>9,000 yuan	1,203 (9.2%)	477 (8.5%)	
Body mass index			0.226
<18.5 kg/m^2^	8,207 (62.9%)	3,575 (63.9%)	
18.5–23.9 kg/m^2^	1,262 (9.7%)	518 (9.3%)	
24–27.9 kg/m^2^	2,961 (22.7%)	1,211 (21.7%)	
>27.9 g/m^2^	623 (4.8%)	288 (5.2%)	
Smoking			0.935
Non-smoker	13,048 (100.0%)	5,590 (100.0%)	
Smoker	5 (0.0%)	2 (0.0%)	
Drinking			0.416
Non-drinker	13,019 (99.7%)	5,581 (99.8%)	
Drinker	34 (0.3%)	11 (0.2%)	
Tea or coffee			0.356
No	8,948 (68.6%)	3,795 (67.9%)	
Yes	4,105 (31.4%)	1,797 (32.1%)	
Milk			0.213
No	4,648 (35.6%)	1,938 (34.7%)	
Yes	8,405 (64.4%)	3,654 (65.3%)	
Menarcheal age			0.883
<12 years	9,910 (75.9%)	4,248 (76.0%)	
12–17 years	2,886 (22.1%)	1,240 (22.2%)	
>17 years	257 (2.0%)	104 (1.9%)	
Length of the menstrual cycle			0.532
<26 days	327 (2.5%)	143 (2.6%)	
26–31 days	10,177 (78.0%)	4,385 (78.4%)	
32–39 days	1,782 (13.7%)	766 (13.7%)	
>39 days	767 (5.9%)	298 (5.3%)	
Regularity of the menstrual cycle			0.224
Within 1–2 days	2,219 (17.0%)	991 (17.7%)	
Within 3–4 days	4,517 (34.6%)	1,950 (34.9%)	
Within 5–7 days	3,097 (23.7%)	1,357 (24.3%)	
> 7 days	1,994 (15.3%)	793 (14.2%)	
Irregular	1,226 (9.4%)	501 (9.0%)	
Menstrual period			0.633
<3 days	682 (5.2%)	283 (5.1%)	
3–7 days	11,012 (84.4%)	4,693 (83.9%)	
>7 days	1,147 (8.8%)	517 (9.2%)	
Irregular	212 (1.6%)	99 (1.8%)	
Menstrual blood flow#			0.750
Less than 1 pack	1,890 (14.5%)	792 (14.2%)	
1 or 2 packs	7,532 (57.7%)	3,219 (57.6%)	
More than 2 packs	3,631 (27.8%)	1,581 (28.3%)	
Number of bleeding between menstruation			0.208
<1 times	10,837 (83.0%)	4,657 (83.3%)	
1–3 times	1,902 (14.6%)	824 (14.7%)	
>3 times	314 (2.4%)	111 (2.0%)	
The degree of dysmenorrhea			0.587
No	2,743 (21.0%)	1,141 (20.4%)	
Melt	6,375 (48.8%)	2,717 (48.6%)	
Moderate	3,007 (23.0%)	1,335 (23.9%)	
Severe	928 (7.1%)	399 (7.1%)	
Number of pregnancies			0.890
0	3,783 (29.0%)	1,638 (29.3%)	
1	3,057 (23.4%)	1,326 (23.7%)	
2	3,322 (25.5%)	1,398 (25.0%)	
> = 3	2,891 (22.1%)	1,230 (22.0%)	
Shift pattern			0.840
Only day shift	4,079 (31.2%)	1,726 (30.9%)	
Two shifts	4,521 (34.6%)	1,973 (35.3%)	
Three shifts	4,245 (32.5%)	1,801 (32.2%)	
Others	208 (1.6%)	92 (1.6%)	
Nap per week			0.471
1–2	4,029 (30.9%)	1,676 (30.0%)	
3–4	3,937 (30.2%)	1,702 (30.4%)	
> = 5	5,087 (39.0%)	2,214 (39.6%)	
Sleep quality			0.967
Well	8,055 (61.7%)	3,449 (61.7%)	
Poor	4,998 (38.3%)	2,143 (38.3%)	
Level of bullying			0.443
No	10,567 (81.0%)	4,529 (81.0%)	
Often	1,579 (12.1%)	699 (12.5%)	
Always	907 (6.9%)	364 (6.5%)	
Level of social support			0.165
Low	2,344 (18.0%)	948 (17.0%)	
Medium	4,251 (32.6%)	1,881 (33.6%)	
High	6,458 (49.5%)	2,763 (49.4%)	
Trait coping style			0.329
Positive	7,533 (57.7%)	3,166 (56.6%)	
Neutral	3,390 (26.0%)	1,474 (26.4%)	
Negative	2,130 (16.3%)	952 (17.0%)	
Anxiety			0.495
No	5,782 (44.3%)	2,434 (43.5%)	
Melt	5,378 (41.2%)	2,328 (41.6%)	
Moderate	1,259 (9.6%)	571 (10.2%)	
Severe	634 (4.9%)	259 (4.6%)	
Depression			0.227
No	5,444 (41.7%)	2,280 (40.8%)	
Melt	5,230 (40.1%)	2,262 (40.5%)	
Moderate	1,096 (8.4%)	453 (8.1%)	
Severe	854 (6.5%)	414 (7.4%)	
Extremely severe	429 (3.3%)	183 (3.3%)	
Physical activity			0.202
Low	4,155 (31.8%)	1,766 (31.6%)	
Medium	2,831 (21.7%)	1,278 (22.9%)	
High	6,067 (46.5%)	2,548 (45.6%)	
Perceived stress			0.199
Low	2,196 (16.8%)	988 (17.7%)	
Medium	10,457 (80.1%)	4,417 (79.0%)	
High	400 (3.1%)	187 (3.3%)	

### 3.2. Variable selection

**Figure 2:**
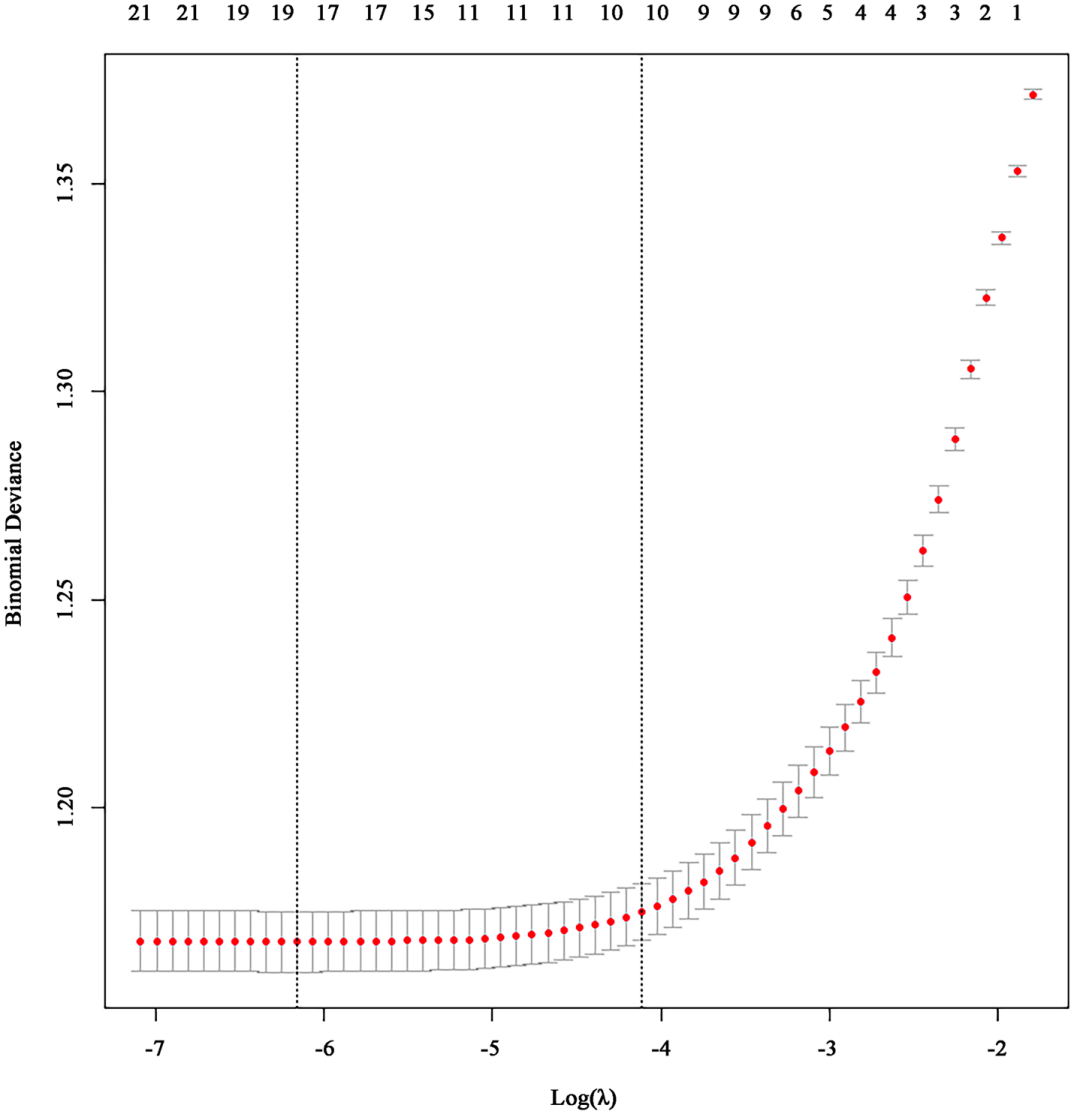
Internal cross-validation of LASSO regression.

We used LASSO regression to select predictive factors associated with PMS. This method brings the advantage of variable selection, adjusts complexity while fitting generalized linear models, and effectively handles collinear data—a pivotal consideration given the substantial number of predictive factors in our study. LASSO regression for internal cross-validation pinpointed 10 variables, as shown in Figure 2, These variables included tea or coffee, sleep quality, regularity of the menstrual cycle, number of bleeding between menstruation, the degree of dysmenorrhea, level of bullying, trait coping style, anxiety, depression and perceived stress.

### 3.3. Construction of predictive models

**Figure 3:**
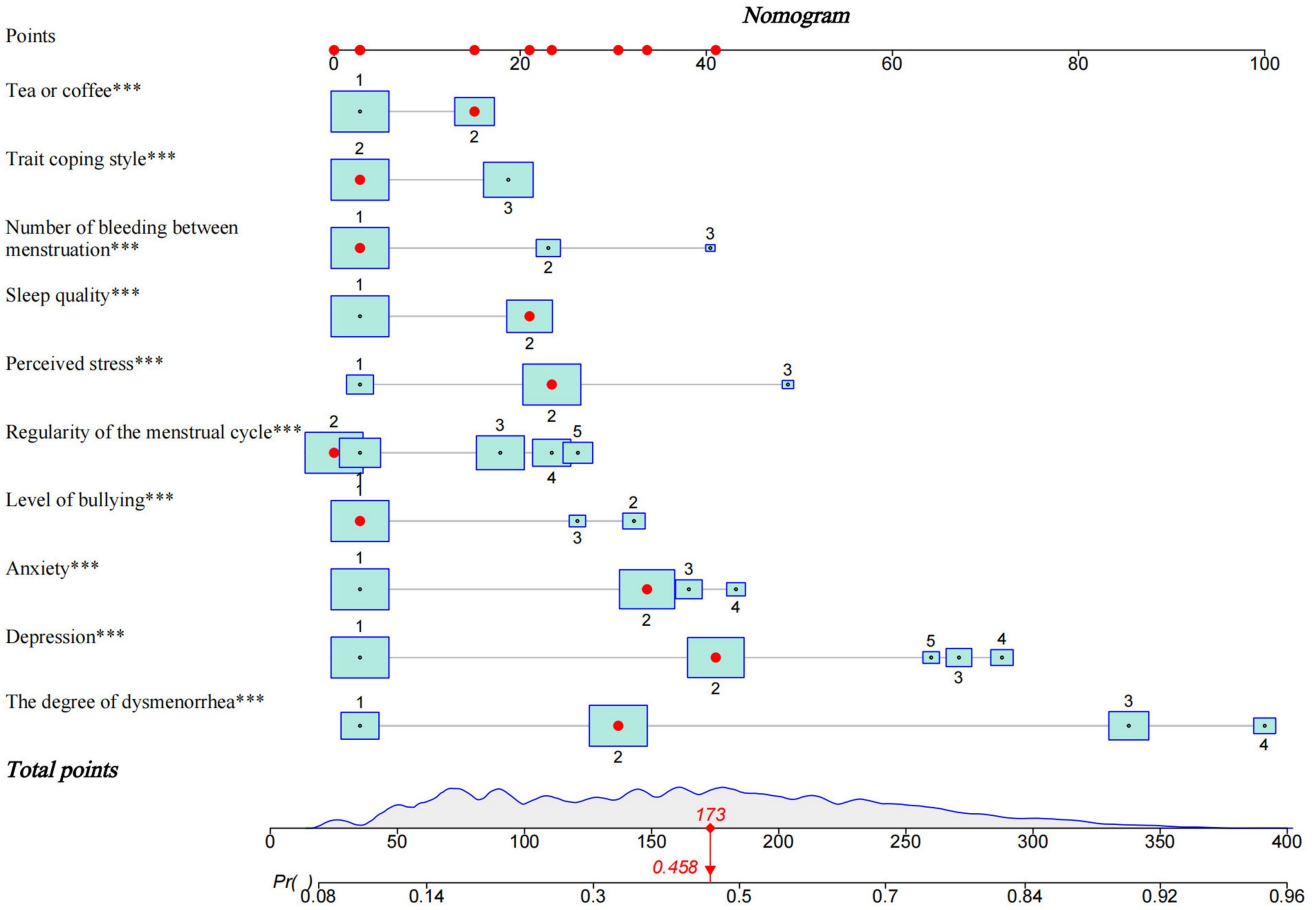
Nomogram model for predicting the risk of PMS in nurses.

The 10 variables selected underwent a multivariate logistic regression analysis with backward stepwise regression. The results underscored the significance of tea or coffee, sleep quality, regularity of the menstrual cycle, number of bleeding between menstruation, the degree of dysmenorrhea, level of bullying, trait coping style, anxiety levels, depression and perceived stress in relation to the occurrence of PMS among nurses (Table 3). Subsequently, a nomogram was constructed based on the results of a multivariate logistic regression model (Figure 3). The nomogram offered an intuitive representation of risk factors for PMS in nurses and facilitated predictions regarding PMS risk. Notably, the severity of dysmenorrhea was assigned the highest risk score, totaling 100 points. To calculate an individual’s risk score, each unique PMS risk factor was traced upwards to the corresponding row on the scale, and the scores for all 10 factors were summated, yielding a total score. Higher total scores indicated an elevated risk of PMS for nurses.

**Table 3:** Stepwise multivariate logistic regression analysis of the training set.

Variables	*β*	SE	Wald values	*p*-value	OR (95% CI)
Physiological factors					
*Regularity of the menstrual cycle (ref: Within 1–2 d)*					
Within 3–4 d	−0.049	0.060	−0.820	0.412	0.952 (0.846–1.071)
Within 5–7 d	0.185	0.064	2.897	0.004	1.204 (1.062–1.365)
More than 7 d	0.340	0.071	4.801	0.000	1.405 (1.223–1.614)
Irregular	0.204	0.082	2.489	0.013	1.226 (1.044–1.439)
*Number of intermenstrual bleeding (ref: <1 times)*					
1–3 times	0.246	0.056	4.353	0.000	1.278 (1.145–1.428)
> 3 times	0.655	0.136	4.796	0.000	1.924 (1.476–2.521)
*The degree of dysmenorrhea (ref: No)*					
Melt	0.396	0.054	7.259	0.000	1.485 (1.335–1.653)
Moderate	1.189	0.063	19.006	0.000	3.283 (2.905–3.712)
Severe	1.410	0.089	15.818	0.000	4.095 (3.441–4.880)
Occupational and behavioral factors					
*Tea or coffee (ref: No)*					
Yes	0.180	0.043	4.200	0.000	1.197 (1.101–1.302)
*Sleep quality (PSQI) (ref: well)*					
Poor	0.323	0.045	7.215	0.000	1.381 (1.265–1.507)
Social and psychological factors					
*Level of bullying (ref: No)*					
Often	0.428	0.063	6.813	0.000	1.535 (1.357–1.737)
Always	0.401	0.086	4.649	0.000	1.493 (1.262–1.740)
*Trait coping style (ref: Negative)*					
Positive	−0.151	0.058	−2.585	0.010	0.860 (0.767–0.964)
Neutral	0.102	0.064	1.591	0.112	1.107 (0.977–0.964)
*Anxiety (ref: No)*					
Melt	0.457	0.058	7.944	0.000	1.579 (1.411–1.768)
Moderate	0.428	0.098	4.352	0.000	1.533 (1.265–1.859)
Severe	0.430	0.151	2.858	0.004	1.538 (1.146–2.069)
*Depression (ref: No)*					
Melt	0.601	0.059	10.218	0.000	1.824 (1.625–2.047)
Moderate	0.903	0.093	9.699	0.000	2.467 (2.056–2.963)
Severe	1.119	0.119	9.414	0.000	3.061 (2.427–3.868)
Extremely severe	0.985	0.178	5.519	0.000	2.677 (1.889–3.804)
*Perceived stress (ref: Low)*					
Medium	0.306	0.061	5.048	0.000	1.358 (1.206–1.530)
High	0.620	0.148	4.189	0.000	1.858 (1.394–2.491)

*β* is the regression coefficient. SE, standard error; OR, odds ratio; CI, confidence interval.

### 3.4. Validation of predictive models

**Figure 4:**
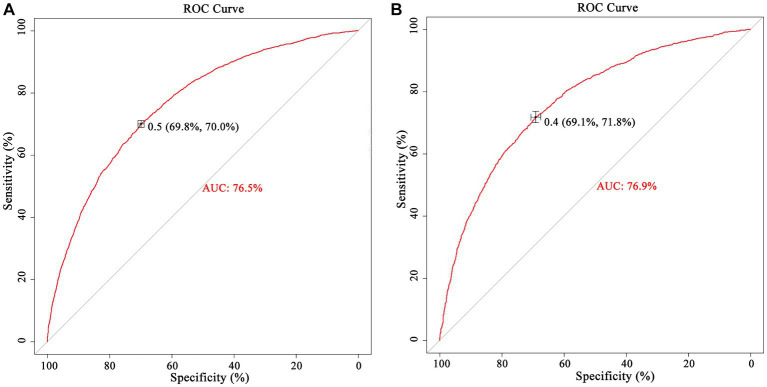
The nomogram model predicts the receiver operating characteristic curve of the PMS in nurse. [(A) training set and (B) test set].

**Figure 5:**
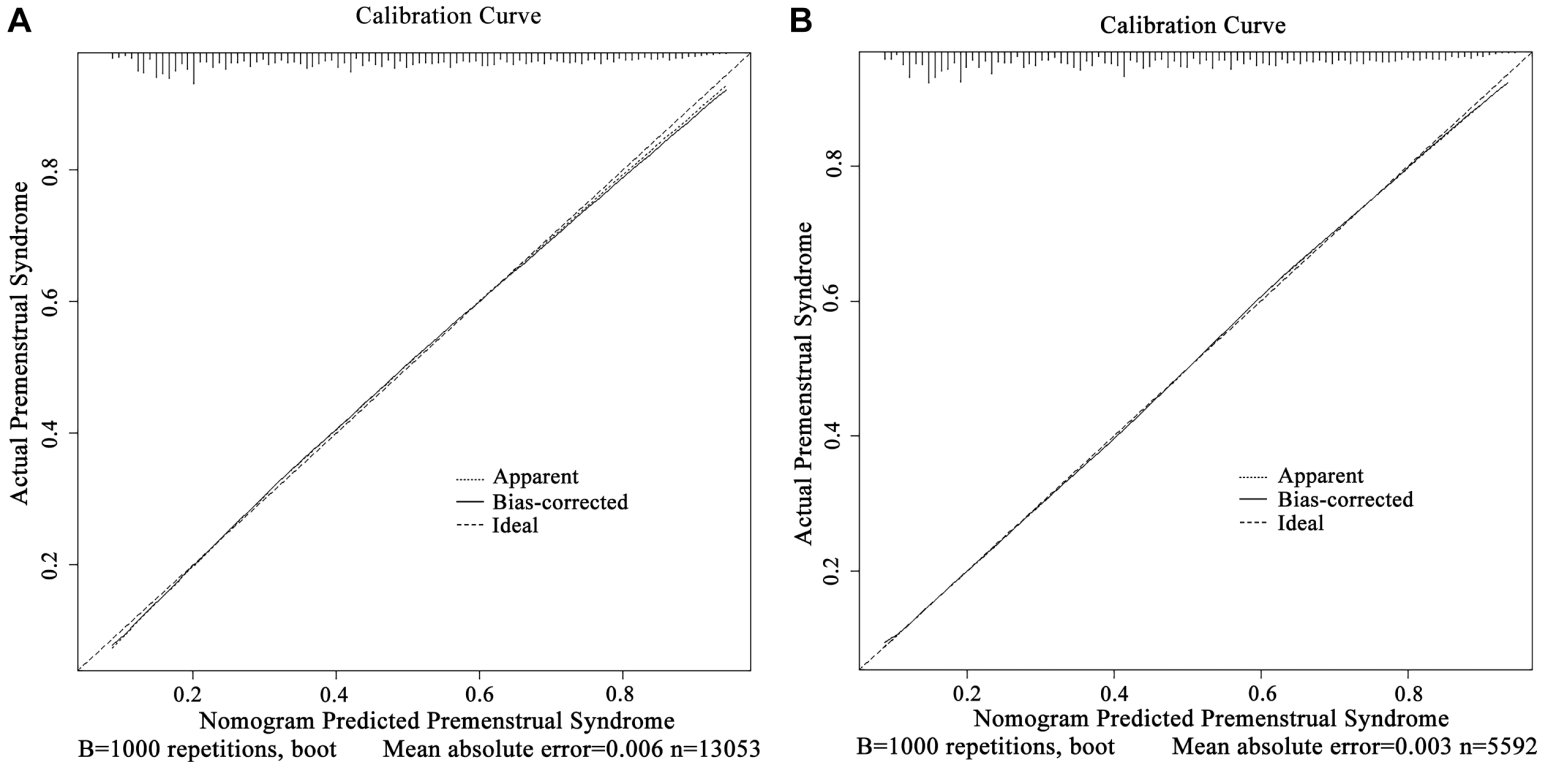
Calibration curve of the nomogram model for predicting PMS in nurses. [(A) training set and (B) test set].

ROC curves were used to evaluate the accuracy of the prediction models. The results revealed that the area under the ROC curve was 0.765 (95% CI, 0.757 ~ 0.773) for the training set and 0.769 (95% CI, 0.756 ~ 0.781) for the test set, respectively (Figures 4A,B). Correspondingly, the C-index was 0.765 and 0.769 for the training and test sets, respectively. The above results affirmed the robust discriminative ability and moderate accuracy of the prediction model. Next, we used calibration curves to evaluate the deviation between the predicted results of the nomogram and the actual values. The prediction results showed a good agreement between the training and test sets (Figures 5A,B). The calibration curves closely paralleled the ideal predictions, yielding mean absolute errors of 0.006 and 0.003 after 1,000 bootstrap repetitions. These outcomes not only establish the model’s dependable calibration but also underscore its reliability.

## 4. Discussion

In this study, a risk prediction model for the PMS among nurses was constructed by multivariate logistic regression, and the nomogram was plotted to visualize the results. The model was validated by ROC curves and calibration curves, revealing close alignment between predicted and observed values and confirming its reliability. Through this modeling process, we identified ten influential risk factors for PMS in nurses, including tea or coffee consumption, sleep quality, regularity of the menstrual cycle, number of bleeding between menstruation, the degree of dysmenorrhea, level of bullying, trait coping style, anxiety, depression and perceived stress. According to the nomogram model, dysmenorrhea was the most significant factor among the ten factors, followed by depression, perceived stress and anxiety.

The results of our study indicated the significant influence of menstrual cycle regularity on PMS. This observation might be elucidated through hormonal mechanisms in the endocrine system. Hormones like progesterone, luteinizing hormone and prolactin play pivotal roles in maintaining normal menstruation in women, and their imbalances could lead to menstrual disorders, which might contribute to the development of PMS or mediate some of the neuropsychiatric symptoms associated with PMS (44–46). In addition, the conclusion of the menstrual cycle aligns with Eshetu’s (15) and Tarannum’s (47) argument that irregular menstrual cycles were a risk factor for PMS.

In terms of menstruation-related factors, our results also revealed a high prevalence of dysmenorrhea (79.2%) among participants with PMS, reaffirming the established association between dysmenorrhea and PMS, while emphasizing the role of dysmenorrhea as the most important risk factor in PMS (14, 48). We also found that the increased frequency of intermenstrual bleeding occurrences was also a risk factor for PMS. This observation may be linked to ovulation-related factors, as bleeding during ovulation often results from a short-term decline in estrogen levels that is insufficient to maintain endometrial stability, leading to small vaginal bleeds during ovulation. At present, its etiology was not clear, hypothalamic–pituitary-ovarian axis disruption may be implicated. However, menarche age was not identified as a PMS risk factor in our study, which is inconsistent with previous research results, which studies students (15, 49), suggesting potential recall bias among participants.

Our study further indicated that nurses who reported coffee or tea consumption were more likely to develop PMS than non-consumers, which also supported previous reports (48). At present, Existing research supports a link between coffee intake and adverse effects on female hormone levels, including lower estrogen and sex hormone binding globulin (SHBG) (50, 51). However, a longitudinal study has yielded opposite results (20), necessitating further investigation into this relationship.

An interesting finding in our study was that shift frequency among nurses was not associated with the occurrence of PMS. While a previous study found that PMS negatively affected work in nurses with three rotating shifts, it did not specifically attribute this to PMS (52). Furthermore, our results highlighted that poor sleep quality, often attributed to hormonal fluctuations and emotional disturbances in women’s menstrual cycles, was a significant PMS risk factor (21, 22, 53). Poor sleep quality leads to fatigue, drowsiness and irritability the next day, which will adversely affect nursing quality.

Our study also illuminated the predictive value of workplace bullying, personality traits, anxiety, depression and perceived stress in relation to PMS. Introverted personalities and occupational stress are more prone to PMS (22, 24, 54). Introverted individuals tend to experience negative emotions such as anxiety, depression, and anger, contributing to PMS development. Elevated levels of fatigue, depressed mood, anxiety and decreased interest in daily activities have been identified as strong PMS predictors (55). Additionally, the autonomic nervous system (ANS) may play a role, as high-intensity cognitive social stress can lead to ANS responses characterized by low arousal and delayed recovery in PMS-affected women. When faced with stress and adverse emotional experiences, their ANS response may render them unable to cope with stress stimuli and emotional experiences (56). However, scant research has explored the impact of workplace bullying on PMS in nurses. The noteworthy discovery that workplace bullying serves as a predictor of PMS diverges from findings in the general population. We hypothesize that association may be attributed to the adverse effects of workplace bullying, such as diminished job satisfaction and the increased possibility of experiencing negative consequences, including depression, stress, and other physical and psychological symptoms, which can contribute to the development of premenstrual syndrome (57, 58). Therefore, future research should delve deeper into the influence of social factors on nurses with PMS.

## 5. Limitations and recommendations

Despite being one of the most extensive studies involving a substantial sample of Chinese nurses and pioneering the exploration of a risk prediction model for PMS in this population, there were some limitations that necessitate consideration. Firstly, our study’s cross-sectional nature precludes the establishment of causal relationships between the identified factors and PMS. To facilitate a more comprehensive understanding of these associations, we recommend future research to prioritize longitudinal investigations. Secondly, reliance on self-reported measurements in this study introduces the potential for recall bias due to the inherent subjectivity of such assessments. Thirdly, the utilization of the number of sanitary napkins as the method for measuring menstrual flow may introduce measurement bias. However, this study offers valuable insights with implications for clinical practice and preventative strategies. We aspire that the observed higher incidence of PMS serves as a catalyst for increased awareness among nurses and healthcare administrators. In addition, we strongly recommend that nurses with menstrual abnormalities can seek medical evaluation and intervention. Finally, we also highly endorse the establishment of nurse counseling centers, the implementation of staff wellness programs, or the integration of virtual reality technology to bolster nurses’ mental health and overall well-being. A healthier nursing workforce is pivotal for ensuring the safety and care quality of patients.

## 6. Conclusion

The prediction model developed in this research demonstrates its potential to effectively predict PMS risk in nurses. The identified risk factors include severe dysmenorrhea, intermenstrual bleeding, irregular menstrual cycle, severe depression and anxiety, high stress, negative coping style, workplace bullying, poorer sleep quality and tea or coffee consumption increased the risk of PMS in nurses. As we move forward, it is crucial to refine the risk prediction model, validate its effectiveness in clinical settings, and continue exploring innovative approaches to support nurses in managing and mitigating the impact of PMS.

## Data availability statement

The data analyzed in this study is subject to the following licenses/restrictions: the data presented in this paper is not readily available. Because the data from the TARGET Nurses’ health cohort study needs time for data clearing and establishment of guidelines. We are planning to make this data available to the public in the future. Requests to obtain data should be sent directly to caoyj@sdu.edu.cn. Requests to access these datasets should be directed to caoyj@sdu.edu.cn.

## Ethics statement

The studies involving humans were approved by the Qilu Hospital of Shandong province’s ethics committee (Approved No. of ethic committee: KYLL-202210-060-1). The studies were conducted in accordance with the local legislation and institutional requirements. The participants provided their written informed consent to participate in this study.

## Author contributions

YC: conceptualization, resources, supervision, and project administration. LL, XL, and YL: methodology and writing-review and editing. XL and YL: project administration and data curation. LL and YL: software. XZ and ML: investigation. LL: writing-original draft preparation, formal analysis, and visualization. All authors have read and agreed to the published version of the manuscript.

## Abbreviations

PMS: Premenstrual Syndrome
PMSS: Premenstrual Syndrome Scale
IPAQ: International Physical Activity Questionnaire
PSQI: Pittsburgh Sleep Quality Index
NAQ-R: Negative Acts Questionnaire-revised
PSSS: Perceived Social Support Scale
PSS: Perceived Stress Scale
SHBG: Sex Hormone Binding Globulin
TCSQ: Trait Coping Style Questionnaire
ANS: Autonomic Nervous System
