# Supplementary material for: Development and validation of risk prediction model for premenstrual syndrome in nurses: results from the nurses-based the TARGET cohort study
Source: Front Public Health. 2023 Oct 3;11:1203280. doi: 10.3389/fpubh.2023.1203280 (PMC10579606; doi:10.3389/fpubh.2023.1203280)
Supplement: Supplementary file 1 [file Data_Sheet_1.PDF]

Supplementary materials

Table 1. Results of Multiple Linear Regression Analysis of Non-Significant Variables in LASSO Regression Models

Variables	*β*	SE	Wald values	*p*-Value	OR (95% CI)
**Age (ref: 31-35 years)**					
20-25 years	0.241	0.105	2.302	0.021	1.273 (1.037–1.564)
26-30 years	0.157	0.071	2.207	0.027	1.170 (1.018–1.346)
36-40 years	-0.104	0.067	-1.566	0.117	0.901(0.791–1.026)
41-45 years	-0.383	0.107	-3.584	0.000	0.682 (0.553–0.840)
**Education level (ref: College degree or below)**					
Under graduate degree or above	0.198	0.085	2.333	0.020	1.219(1.033–1.440)
**Marital status (ref: Unmarried)**					
Married	0.103	0.090	1.150	0.250	1.109 (0.930–1.321)
Others	0.233	0.184	1.264	0.206	1.262 (0.879–1.809)
**Work experience (ref: 0-5 years)**					
6-10 years	-0.028	0.070	-0.392	0.695	0.973 (0.848–1.116)
11-15 years	0.073	0.084	0.872	0.383	1.076 (0.913–1.268)
>15 years	0.214	0.109	1.968	0.049	1.239(1.001–1.533)
**Monthly income (ref: <3000 yuan)**					
3000-6000 yuan	0.025	0.079	0.316	0.752	1.025 (0.878–1.198)
6000-9000 yuan	0.086	0.086	1.002	0.316	1.090 (0.921–1.291)
>9000 yuan	0.096	0.104	0.928	0.354	1.101 (0.898–1.350)
**Body mass index (ref: 18.5-23.9 kg/m^2)**					
<18.5 kg/m^2	-0.048	0.070	-0.681	0.496	0.953 (0.831–1.094)
24-27.9 kg/m^2	0.027	0.051	0.539	0.590	1.028 (0.930–1.136)
>27.9 kg/m^2	0.077	0.097	0.797	0.425	1.090 (0.893–1.306)
**Smoking (ref: Non-smoker)**					
Smoker	-0.853	0.972	-0.877	0.381	0.426 (0.051–2.824)
**Drinking (ref: Non-drinker)**					
Drinker	-0.985	0.444	-2.218	0.027	0.373 (0.154–0.887)
**Milk (ref: No)**					
Yes	0.000	0.046	-0.004	0.996	0.999 (0.913–1.095)
**Physical Activity (ref: Low)**					
Medium	-0.152	0.058	-2.620	0.009	0.859 (0.767–0.962)
High	-0.055	0.049	-1.135	0.256	0.946 (0.861–1.041)
**Menarcheal age (ref: 12-17 years)**					
<12 years	0.036	0.049	0.730	0.465	1.037 (0.941–1.141)
17 years	-0.155	0.146	-1.060	0.289	0.856 (0.642–1.140)
**Length of the menstrual cycle (ref: <21 days)**					
22-31days	-0.207	0.135	-1.533	0.125	0.813 (0.624–1.059)
32-39 days	-0.262	0.143	-1.825	0.068	0.770 (0.581–1.019)
>39 days	-0.220	0.158	-1.394	0.163	0.803 (0.589–1.093)
**Menstrual period (ref: 3-7 days)**					

<3 days	0.216	0.097	2.222	0.026	1.241 (1.026–1.501)
>7 days	0.153	0.074	2.070	0.038	1.166 (1.008–1.348)
Irregular	0.075	0.170	0.444	0.657	1.078 (0.774–1.508)
**Menstrual blood flow (ref: 1 or 2 packs)**					
Less than 1 pack	-0.113	0.063	-1.781	0.075	0.893 (0.789–1.011)
More than 2 packs	0.041	0.048	0.867	0.386	1.042 (0949–1.145)
**Number of pregnancies (ref: 0 time)**					
1time	-0.025	0.084	-0.300	0.764	0.975 (0.828–1.149)
2 times	-0.030	0.086	-0.347	0.728	0.971(0.820–1.149)
>=3 times	0.110	0.090	1.226	0.220	1.116 (0.936–1.331)
**Shift pattern (ref: Only day shift)**					
Two shifts	-0.116	0.054	-2.145	0.032	0.891 (0.801–0.990)
Three shifts	-0.142	0.055	-2.586	0.010	0.867 (0.778–0.966)
Others	0.039	0.167	0.234	0.815	1.040 (0.750–1.443)
**Nap per week (ref: 1-2 times)**					
3-4 times	0.048	0.052	0.924	0.356	1.050 (0.947–1.163)
>=5 times	-0.052	0.051	-1.024	0.306	0.950 (0.860–1.048)
**Level of social support (ref: Low)**					
Medium	-0.025	0.063	-0.403	0.687	0.975 (0.862–1.102)
High	-0.049	0.063	-0.777	0.437	0.952 (0.841–1.078)
