# Supplementary material for: Development and validation of risk prediction model for premenstrual syndrome in nurses: results from the nurses-based the TARGET cohort study
Source: Front Public Health. 2023 Oct 3;11:1203280. doi: 10.3389/fpubh.2023.1203280 (PMC10579606; doi:10.3389/fpubh.2023.1203280)
Supplement: Supplementary file 2 [file Data_Sheet_2.PDF]

Questionnaire (English version)

1. Self-designed questionnaires

1. Date of birth: ______Y-______M-______D	
2. Height: ________cm	
3. Weight: ________kg	
4. Time of participation: ________Y	
**Questions**	**Options**
5. Highest educational level	a. College degree or below<br>b. Undergraduate degree or above
6. Current average monthly income	a. <3000 yuan<br>b. 3000–6000 yuan<br>c. 6000–9000 yuan<br>d. >9000 yuan
7. Present marital status	a. Unmarried b. Married c. Others
8. On average, how many days a week have you napped in the past year?	a. 1-2 times/w b. 3-4 times/w c. >= 5 times/w
9. Are you currently a tea drinker in the past year?	a.No b. Yes
10. Are you currently a coffee drinker in the past year?	a.No b. Yes
11. Are you currently a smoker in the past year?	a.No b. Yes
12. Are you currently drinking alcohol in the past year?	a.No b. Yes
13. Do you have the habit of drinking milk in the past year?	a.No b. Yes
14. Age of first menstruation	a. < 12 years b. 12–17 years c. > 17 years
15. Menstrual cycle: how many days does it usually take from the first day of one menstrual cycle to the first day of the next?	a. < 26 days<br>b. 26–31 days<br>c. 32–39 days<br>d. > 39 days
16. Have you had regular menstrual cycles for the past 6 months?	a. Within 1–2 days<br>b. Within 3–4 days<br>c. Within 5–7 days<br>d. > 7 days<br>e. Irregular
17. How many days are your periods (duration of each period)?	a. < 3 days<br>b. 3–7 days<br>c. > 7 days<br>d. irregular
18. What is the number of sanitary napkins you use for each menstrual period? (Each pack is based on 10 tablets)	a. less than 1 pack of sanitary napkins<br>b. 1 or 2 packs of sanitary napkins<br>c. more than 2 packs of sanitary napkins
19. In the past year, how many times have you had bleeding or spotting (other than bleeding after intercourse) between normal menstrual periods?	a. < 1 time b. 1–3 times c.> 3 times
20. In the past year, what has been the severity of menstrual cramps?	a. None b. Mild c. Moderate d. Severe
21. How many pregnancies have you had (including live births, stillbirths, miscarriages)?	a. None b. 1 time c. 2 times d. ≥ 3 times

2. Premenstrual Syndrome Scale (PMS)

Did you experience any of the following symptoms during the 14 days prior to your last menstrual period through your period?				
**Symptoms**	**No symptoms**	**Mild symptoms**	**tolerable symptoms affect study, work and life**	**symptoms seriously affect study, work and life, and require treatment**
1. Irritability				
2. Depression				
3. Anxiety				
4. Inattention				
5. Nervousness				
6. Fidgeting				
7. Neuroticism				
8. Bloating				
9. Diarrhea				
10. Sleepiness				
11. Migraine				
12. Insomnia				
13. Swelling of hands and feet				

3. International Physical Activity Questionnaire (IPAQ)

We are interested in finding out about the kinds of physical activities that people do as part of their everyday lives. The questions will ask you about the time you spent being physically active in the **last 7 days**. Please answer each question even if you do not consider yourself to be an active person. Please think about the activities you do at work, as part of your house and yard work, to get from place to place, and in your spare time for recreation, exercise or sport.

Think about all the **vigorous** and **moderate** activities that you did in the **last 7 days**. **Vigorous** physical activities refer to activities that take hard physical effort and make you breathe much harder than normal. **Moderate** activities refer to activities that take moderate physical effort and make you breathe somewhat harder than normal.

PART 1: JOB-RELATED PHYSICAL ACTIVITY

The first section is about your work. This includes paid jobs, farming, volunteer work, course work, and any other unpaid work that you did outside your home. Do not include unpaid work you might do around your home, like housework, yard work, general maintenance, and caring for your family. These are asked in Part 3.

1.Do you currently have a job or do any unpaid work outside your home?

☐ Yes 有

☐ No 没有 ➞ ***Skip to PART 2: TRANSPORTATION***

The next questions are about all the physical activity you did in the **last 7 days** as part of your paid or unpaid work. This does not include traveling to and from work.

2. During the **last 7 days**, on how many days did you do **vigorous** physical activities like heavy lifting, digging, heavy construction, or climbing up stairs **as part of your work**? Think about only those physical activities that you did for at least 10 minutes at a time. _____ **days per week**

☐ No vigorous job-related physical activity ➞ ***Skip to question 4***

3.How much time did you usually spend on one of those days doing **vigorous** physical activities as part of your work?

_____ **hours per day** _____ **minutes per day**

4.Again, think about only those physical activities that you did for at least 10 minutes at a time. During the **last 7 days**, on how many days did you do **moderate** physical activities like carrying light loads **as part of your work**? Please do not include walking. _____ **days per week 每周几天**

☐ No moderate job-related physical activity ➞ ***Skip to question 6***

5.How much time did you usually spend on one of those days doing **moderate** physical activities as part of your work? _____**hours per day** _____**minutes per day**

6.During the **last 7 days**, on how many days did you **walk** for at least 10 minutes at a time **as part of your work**? Please do not count any walking you did to travel to or from work. _____ **days per week**

☐ No job-related walking ➞ ***Skip to PART 2: TRANSPORTATION***

7. How much time did you usually spend on one of those days **walking** as part of your work?

_____ **hours per day** _____ **minutes per day**

PART 2: TRANSPORTATION PHYSICAL ACTIVITY

These questions are about how you traveled from place to place, including to places like work, stores, movies, and so on.

8.During the **last 7 days**, on how many days did you **travel in a motor vehicle** like a train, bus, car, or tram?

_____ **days per week**

☐ No traveling in a motor vehicle ➞ ***Skip to question 10***

9.How much time did you usually spend on one of those days **traveling** in a train, bus, car, tram, or other kind of motor vehicle?

_____ **hours per day** _____ **minutes per day**

Now think only about the **bicycling** and **walking** you might have done to travel to and from work, to do errands, or to go from place to place.

10.During the **last 7 days**, on how many days did you **bicycle** for at least 10 minutes at a time to go **from place to place**?

_____ **days per week**

☐ No bicycling from place to place ➞ ***Skip to question 12***

11.How much time did you usually spend on one of those days to **bicycle** from place to place?

_____ **hours per day** _____ **minutes per day**

12.During the **last 7 days**, on how many days did you **walk** for at least 10 minutes at a time to go **from place to place**?

_____ **days per week**

☐ No walking from place to place ➞ ***Skip to PART 3: HOUSEWORK, HOUSE MAINTENANCE, AND CARING FOR FAMILY***

13.How much time did you usually spend on one of those days **walking** from place to place?

_____ **hours per day** _____ **minutes per day**

PART 3: HOUSEWORK, HOUSE MAINTENANCE, AND CARING FOR FAMILY

This section is about some of the physical activities you might have done in the **last 7 days** in and around your home, like housework, gardening, yard work, general maintenance work, and caring for your family.

14.Think about only those physical activities that you did for at least 10 minutes at a

time. During the **last 7 days**, on how many days did you do **vigorous** physical activities like heavy lifting, chopping wood, shoveling snow, or digging **in the garden or yard**?

_____ **days per week**

☐ No vigorous activity in garden or yard → ***Skip to question 16***

15. How much time did you usually spend on one of those days doing **vigorous** physical activities in the garden or yard?

_____ **hours per day** _____ **minutes per day**

16. Again, think about only those physical activities that you did for at least 10 minutes at a time. During the **last 7 days**, on how many days did you do **moderate** activities like carrying light loads, sweeping, washing windows, and raking **in the garden or yard**?

_____ **days per week**

☐ No moderate activity in garden or yard → ***Skip to question 18***

17. How much time did you usually spend on one of those days doing **moderate** physical activities in the garden or yard?

_____ **hours per day** _____ **minutes per day**

18. Once again, think about only those physical activities that you did for at least 10 minutes at a time. During the **last 7 days**, on how many days did you do **moderate** activities like carrying light loads, washing windows, scrubbing floors and sweeping **inside your home**?

_____**days per week**

☐ No moderate activity inside home → ***Skip to PART 4: RECREATION, SPORT AND LEISURE-TIME PHYSICAL ACTIVITY***

19. How much time did you usually spend on one of those days doing **moderate** physical activities inside your home?

_____ **hours per day** _____ **minutes per day**

PART 4: RECREATION, SPORT, AND LEISURE-TIME PHYSICAL ACTIVITY

This section is about all the physical activities that you did in the **last 7 days** solely for recreation, sport, exercise or leisure. Please do not include any activities you have already mentioned.

20. Not counting any walking you have already mentioned, during the **last 7 days**, on how many days did you **walk** for at least 10 minutes at a time **in your leisure time**?

_____ **days per week**

☐ No walking in leisure time → ***Skip to question 22***

21. How much time did you usually spend on one of those days **walking** in your

leisure time?

_____ **hours per day** _____ **minutes per day**

22. Think about only those physical activities that you did for at least 10 minutes at a time. During the **last 7 days**, on how many days did you do **vigorous** physical activities like aerobics, running, fast bicycling, or fast swimming **in your leisure time**?

_____ **days per week**

☐ No vigorous activity in leisure time → ***Skip to question 24***

23. How much time did you usually spend on one of those days doing **vigorous** physical activities in your leisure time?

_____ **hours per day** _____**minutes per day**

24. Again, think about only those physical activities that you did for at least 10 minutes at a time. During the **last 7 days**, on how many days did you do **moderate** physical activities like bicycling at a regular pace, swimming at a regular pace, and doubles tennis **in your leisure time**?

_____ **days per week**

☐ No moderate activity in leisure time → ***Skip to PART 5: TIME SPENT SITTING***

25. How much time did you usually spend on one of those days doing **moderate** physical activities in your leisure time?

_____ **hours per day** _____ **minutes per day**

PART 5: TIME SPENT SITTING

The last questions are about the time you spend sitting while at work, at home, while doing course work and during leisure time. This may include time spent sitting at a desk, visiting friends, reading or sitting or lying down to watch television. Do not include any time spent sitting in a motor vehicle that you have already told me about.

26. During the **last 7 days**, how much time did you usually spend **sitting** on a **weekday**?

_____ **hours per day** _____**minutes per day**

27. During the **last 7 days**, how much time did you usually spend **sitting** on a **weekend day**?

_____ **hours per day** _____**minutes per day**

4. **The Pittsburgh Sleep Quality Index (PSQI)**

Instructions: The following questions relate to your usual sleep habits during the past month only. Your answers should indicate the most accurate reply for the majority of days and nights in the past month. Please answer all questions.

During the past month,

1. When have you usually gone to bed?____________
2. How long (in minutes) has it taken you to fall asleep each night?______________
3. When have you usually gotten up in the morning?_______________
4. How many hours of actual sleep do you get at night? (This may be different than the number of hours you spend in bed)___________

5. During the past month, how often have you had trouble sleeping because you..	Not during the past month (0)	Less than once a week (1)	Once or twice a week (2)	Three or more times a week (3)
a. Cannot get to sleep within 30 minutes				
b. Wake up in the middle of the night or early morning				
c. Have to get up to use the bathroom				
d.Cannot breathe comfortably				
e. Cough or snore loudly				
f. Feel too cold				
g. Feel too hot				
h. Have bad dreams				
i. Have pain				
j. Other reason(s), please describe, including how often you have had trouble sleeping because of this reason(s):				
6. During the past month, how often have you taken medicine (prescribed or"over the counter") to help you sleep?				
7. During the past month, how often have you had trouble staying awake while driving, eating meals, or engaging in social activity?				
8. During the past month, how much of a problem has it been for you to keep up enthusiasm to get things done?				
	Very good (0)	Fairly good (1)	Fairly bad (2)	Very bad (3)
9. During the past month, how would you rate your sleep quality overall?				

5. The Negative Acts Questionnaire-Revised (NAQ-R)

	Never (1)	Occasionally (occurs more than once a month) (2)	Monthly (occurs at least 1 time per month) (3)	Every week (at least 1 time per week) (4)	Every day (at least 1 time per day) (5)
1. Your job performance has been affected by a coworker withholding information from you					
2. Placing you in jobs below your level of competence.					
3. Depriving you of your primary job duties and placing you in menial and unpleasant tasks.					
4. Gossip is spread about you.					
5. You are ignored, ostracized or isolated by other co-workers.					
6. Your personal circumstances such as habits, background, attitudes or private life are insulted or attacked.					
7. A coworker yells at you in anger and uses you as a punching bag.					
8. Coercive behavior: pointing fingers, invading your personal space, pushing, pulling, dragging, getting in the way, etc.					
9. A coworker implies that you should be transferred from your current job.					
10. A coworker repeatedly mentions your faults or mistakes.					
11. You are treated with coldness or hostility by a coworker.					
12. Repeated criticism of your work and efforts.					
13. Your ideas and opinions are ignored.					
14. You are teased by a coworker with whom you do not have a good relationship.					
15. You are assigned work that is unreasonable, unachievable or not on schedule.					
16. You are criticized by a colleague.					
17. Your work is over-supervised.					
18. Your rights are not guaranteed (e.g. sick leave, statutory breaks,					

travel allowances).					
19. You are the target of teasing and sarcasm from your colleagues.					
20. You are overloaded with work.					
21. You are threatened with violence or bodily harm.					
22. You have been bullied in the workplace in the last six months.					

6. The Perceived Social Support Scale (PSSS)

	Strongly disagree (1)	Highly disagree (2)	Slightly disagree (3)	Neutral (4)	Slightly agree (5)	Strongly agree (6)	Highly agree (6)
1. There are people (leaders, relatives, coworkers) who are there for me when I have problems.							
2. There are people (leaders, relatives, coworkers) with whom I can share my joys and sorrows.							
3. My family is able to help me in practical and concrete ways.							
4. I get emotional help and support from my family when I need it.							
5. There are people (leaders, relatives, coworkers) who are a real source of comfort when I am in trouble.							
6. My friends can really help me.							
7. I can count on my friends in times of trouble.							
8. I can talk to my family about my problems.							
9. My friends can share my joys and sorrows with me.							
10. There are people in my life (leaders, relatives, coworkers) who care about my feelings.							
11. My family willingly assists me in making decisions.							
12. I can discuss my problems with my friends.							

7. The Trait Coping Style Questionnaire (TCSQ)

	Definitely not (1)	Mostly not (2)	Usually yes (3)	Mostly yes (4)	Definitely yes (5)
1. Able to forget unpleasantness as quickly as possible.					
2. Tendency to get caught up in memories and fantasies of events from which one cannot escape.					
3. Act as if the event never happened.					
4. Tend to be angry with others and lose temper frequently.					
5. Usually looks on the bright side and thinks positively.					
6. Easily gets emotional about unpleasant things.					
7. Prefers to keep his/her emotions to himself/herself and not show them, but can't forget them.					
8. Usually feels nothing when compared to similar people.					
9. Can quickly turn negative factors into positive ones, such as participating in activities.					
10. It's easy to cry quietly when you're upset.					
11. It is easy to be cheered up by others.					
12. If you have a conflict with someone, you prefer to ignore them for a long time.					
13. Tend to be indecisive and unable to think of solutions to major difficulties.					
14. Adapt quickly to difficulties and pain.					
15. Believes that difficulties and setbacks can be a good exercise.					
16. Recalls unpleasant encounters over a long period of time.					
17. Tends to blame himself for his own incompetence when he encounters difficult problems.					
18. Believes that there is no big deal in the world.					
19. Prefers to be alone when in distress.					
20. Usually uses humor to resolve embarrassing situations.					

8. Perceived Stress Scale (PSS)

	Never (0)	Occasionally (1)	Sometimes (2)	Often (3)	Always (4)
1. In the last month, how often have you been upset because of something that happened unexpectedly?					
2. In the last month, how often have you felt that you were unable to control the important things in your life?					
3. In the last month, how often have you felt nervous and "stressed"?					
4. In the last month, how often have you felt confident about your ability to handle your personal problems?					
5. In the last month, how often have you felt that things were going your way?					
6. In the last month, how often have you found that you could not cope with all the things that you had to do?					
7. In the last month, how often have you been able to control irritations in your life?					
8. In the last month, how often have you felt that you were on top of things?					
9. In the last month, how often have you been angered because of things that happened that were outside of your control?					
10. In the last month, how often have you felt difficulties were piling up so high that you could not overcome them?					

9. The Generalized Anxiety Disorder 7-Item Scale (GAD-7)

Over the last 2 weeks, how often have you been bothered by the following problems?

	Not at all (0)	Several Days (1)	Over Half The Days (2)	Nearly Everyday (3)
1. Feeling nervous, anxious, or on edge.				
2. Not being able to stop or control worrying.				
3. Worrying too much about different things.				
4. Trouble relaxing.				
5. Being so restless that it's hard to sit still.				
6. Becoming easily annoyed or irritable.				
7. Feeling afraid as if something awful might happen.				

10. The 9-item Patient Health Questionnaire (PHQ-9)

Over the past 2 weeks, how often have you been bothered by any of the following problems?

	Not at all (0)	Several Days (1)	Over Half The Days (2)	Nearly Everyday (3)
1. Little interest or pleasure in doing things.				
2. Feeling down, depressed or hopeless.				
3. Trouble falling asleep, staying asleep, or sleeping too much.				
4. Feeling tired or having little energy				
5. Poor appetite or overeating.				
6. Feeling bad about yourself - or that you're a failure or have let yourself or your family down.				
7. Trouble concentrating on things, such as reading the newspaper or watching television.				
8. Moving or speaking so slowly that other people could have noticed. Or, the opposite - being so fidgety or restless that you have been moving around a lot more than usual.				
9. Thoughts that you would be better off dead or of hurting yourself in some way.				
