# Supplementary material for: Development and validation of risk prediction model for premenstrual syndrome in nurses: results from the nurses-based the TARGET cohort study
Source: Front Public Health. 2023 Oct 3;11:1203280. doi: 10.3389/fpubh.2023.1203280 (PMC10579606; doi:10.3389/fpubh.2023.1203280)
Supplement: Supplementary file 3 [file Data_Sheet_3.PDF]

Questionnaire (Chinese version)

1. Self-designed questionnaires

1. 您的出生时间: ______年-______月-______日	
2. 您的身高: ______cm	
3. 您的体重: ______kg	
4. 您参加工作时间: ______年	
**调查内容**	
5. 您的最高受教育水平	a. 大学学历及以下<br>b. 本科及以上学历
6. 您当前的月收入	a. <3000 元<br>b. 3000–6000 元<br>c. 6000–9000 元<br>d. >9000 元
7. 您当前的婚姻状况	a. 未婚 b. 已婚 c.其他
8. 过去一年中，平均每周有几天小睡？	a. 1-2 次/周 b. 3-4 次/周 c. >= 5 次/周
9. 过去一年，您是否有饮茶习惯？	a.否 b. 是
10. 过去一年，您是否有饮咖啡习惯？	a.否 b. 是
11. 过去一年，您是否吸烟？	a.否 b. 是
12. 过去一年，您是否饮酒？	a.否 b. 是
13. 过去一年，您是否有喝牛奶习惯？	a.否 b. 是
14. 您的月经初潮年龄	a. < 12 岁 b. 12–17 岁 c. > 17 岁
15. 月经周期：从一个月经周期的第一天到下一个月经周期的第一天通常需要多少天?(出血的第一日为月经周期的开始)	a. < 26 天<br>b. 26–31 天<br>c. 32–39 天<br>d. > 39 天
16. 在过去 6 个月，您的月经周期是否规律（从一个周期开始到下一个周期的天数是否与预期的一样)?	a. 1–2 天<br>b. 3–4 天<br>c. 5–7 天<br>d. > 7 天<br>e. 不规律
17. 您的经期（每次月经持续时间)为多少天？	a. < 3 天<br>b. 3–7 天<br>c. > 7 天<br>d. 不规律
18. 您每次月经使用的卫生巾的数量是多少？（每包按 10 片计算）	a. <1 包<br>b. 1-2 包<br>c. >2 包
19. 过去一年，您在两次正常月经之间出现出血或点滴出血(性交后出血除外)的次数?	a. < 1 次 b. 1–3 次 c. > 3 次
20. 过去一年，您是否有痛经(痛经指月经前后或经期出现下腹疼痛、坠胀，伴腰酸或其他不适)？	a. 无 b. 轻度 c. 中度 d. 重度
21. 您怀孕的次数（包括活产、死产、流产）	a. 无 b. 1 次 c. 2 次 d. ≥ 3 次

2. Premenstrual Syndrome Scale (PMS)

症状	无症状	症状轻微	症状影响工作、生活、学习，但能忍受	症状严重影响工作、学习和生活，需要治疗
1. 末次月经前 14 天至经期，您是否出现以下症状？_易激动				
2. 末次月经前 14 天至经期，您是否出现以下症状？_抑郁				
3. 末次月经前 14 天至经期，您是否出现以下症状？_焦虑				
4. 末次月经前 14 天至经期，您是否出现以下症状？_腹胀腹泻				
5. 末次月经前 14 天至经期，您是否出现以下症状？_注意力不集中				
6. 末次月经前 14 天至经期，您是否出现以下症状？_嗜睡				
7. 末次月经前 14 天至经期，您是否出现以下症状？_紧张				
8. 末次月经前 14 天至经期，您是否出现以下症状？_坐立不安				
9. 末次月经前 14 天至经期，您是否出现以下症状？_偏头痛				
10. 末次月经前 14 天至经期，您是否出现以下症状？_失眠				
11. 末次月经前 14 天至经期，您是否出现以下症状？_手脚肿胀				
12. 末次月经前 14 天至经期，您是否出现以下症状？_神经质				
13. 末次月经前 14 天至经期，您是否出现以下症状？_易激动				

3. International Physical Activity Questionnaire (IPAQ)

在下列问题中，重体力活动是指需要您花费大力气完成，呼吸较平常明显增强的活动。中等强度体力活动是指需要您花费中等力气完成，呼吸较平常稍微增强的活动.

第一部分:工作有关体力活动
本部分是询问您的工作情况，这里的工作包括有酬工作，农活和其他您在家庭外从事的无偿工作。不要包括家务劳动，这方面的问题将在第三部分问及。

1. 1a 您现在在职或在家庭外从事无偿工作吗?［单选题］*️

○是　　○否（请跳至第 8 题）

下面的问题是关于您在过去 7 天中进行的有酬或无偿工作中的体力活动，这不包括您在上下班路上的体力活动情况。请只考虑那些每次至少十分钟的体力活动。

2. 1b 过去 7 天，您有几天在工作中进行重体力活动，例如搬(举)、抬、翻重物或患者， 装卸货物、上楼梯等?(只计算那些每次超过 10 分钟的活动)［单选题］*仅填写数字

○没有（0 天）（请跳至第 4 题）　　○有

3. 1c 您在工作中进行这些重体力活动的时间
一周___天　　平均每天___分钟［填空题］*

4. 1d 过去 7 天，您有几天在工作中进行中等强度体力活动，例如搬（举）轻物?不要包括走路。(只计算那些每次超过 10 分钟的活动）［单选题］*

○没有（0 天）（请跳至第 6 题）　　○有

5. 1e 您在工作中进行这些中等强度体力活动的时间
一周___天　　平均每天___分钟［填空题］*

6. 1f 过去 7 天，过去 7 天，您有几天在工作中每次步行超过 10 分钟?（不包括上下班路上的步行）［单选题］*

○没有(0 天)（请跳至第 8 题）　　○有

7. 1g 您平均在工作中步行的时间
一周___天　　平均每天___分钟［填空题］*

第二部分：交通行程有关体力活动
本部分的问题是关于您交通行程的体力活动，包括上下班、购物、买菜等。

8. 2a 过去 7 天，您有几天乘机动车，如火车、公共汽车、电车、私家车、出租车、地铁?［单选题］*

○没有(0)（请跳至第 10 题）　　○有

9. 2b 您平均的乘车的时间
一周___天　　平均每天___分钟［填空题］*

现在只考虑骑自行车和步行。

10. 2c 过去 7 天，您有几天骑自行车（每次超过 10 分钟）？［单选题］*

○没有(0 天)（请跳至第 12 题）　　○有

11. 2d 您平均骑车的时间为
一周___天　平均每天___分钟［填空题］*

12. 2e 过去 7 天，过去 7 天，您有几天步行上下班（每次超过 10 分钟）？[单选题] *

○没有(0 天) (请跳至第 14 题)　　　　　　　　○有

13. 2f 您平均步行的时间为
一周___天　　平均每天___分钟 [填空题] *

第三部分：家务有关体力活动
本部分是关于您的家务劳动情况。请只考虑那些每次至少十分种的体力活动。

14. 3a 过去 7 天，您有几天在户外进行重体力活动，例如搬(举)重物?(只计算每次超过 10 分钟的活动)？ [单选题] *

○没有(0 天) (请跳至第 16 题)　　　　　　　　○有

15. 3b 您平均在院子里进行重体力活动的时间为
一周___天　　平均每天___分钟 [填空题] *

16. 3c 过去 7 天，您有几天在户外进行中等强度体力活动，如搬(举)轻物，遛狗? (只计算每次超过 10 分钟的活动)？ [单选题] *

○没有(0 天) (请跳至第 18 题)　　　　　　　　○有

17. 3d 您平均在院子里进行中等强度体力活动的时间为
一周___天　　平均每天___分钟 [填空题] *

18. 3e 过去 7 天，您在闲暇时有几天进行重体力活动，如跑步，游泳，踢足球、打篮球、打网球、跳绳、健身房内健身等? (只计算每次超过 10 分钟的活动) [单选题] *

○没有(0 天) (请跳至第 20 题)　　　　　　　　○有

19. 3f 您平均在室内进行中等强度体力活动的时间为
一周___天　　平均每天___分钟[填空题] *

第四部分:闲暇时间的体力活动
本部分是关于您在过去 7 天中在闲暇时进行的体育锻炼或运动，只考虑那些每次至少十分种的体力活动。请不要包括那些您在上面已经回答过的体力活动。

20. 4a 不包括您前面已经提过的步行，过去 7 天，您在闲暇时有几天一次步行超过 10 分钟? [单选题] *

○没有(0 天) (请跳至第 22 题)　　　　　　　　○有

21. 4b 您平均步行的时间为
一周___天　　平均每天___分钟[填空题] *

22. 4c 过去 7 天，您在闲暇时有几天进行重体力活动，如跑步，游泳，踢足球、打篮球、打网球、跳绳、健身房内健身等? (只计算每次超过 10 分钟的活动) [单选题] *

○没有(0 天) (请跳至第 24 题)　　　　　　　　○有

23. 4d 您平均闲暇时进行重体力活动的时间为
一周___天　　平均每天___分钟 [填空题] *

24. 4f 过去 7 天，您闲暇时有几天进行中等强度体力活动，如打太极拳、快走、打乒乓球，打羽毛球，跳交谊舞等?(只计算每次超过 10 分钟的活动) [单选题] *

○没有(0 天) (请跳至第 26 题)　　　　　　　　○有

25. 4g 您平均闲暇时进行中等强度体力活动的时间为
一周___天　　平均每天___分钟 [填空题] *

第五部分:静坐时间
最后的问题是关于您处于静坐的时间，包括您在工作单位和家中，坐在办公桌前，电脑前，坐着或躺着看电视，拜访朋友和看书等的时间，但不要包括坐在车里的时间。

26. 5a 过去 7 天，您的工作日有___天，休息日有___天 [填空题] *

27. 5b 过去 7 天，在每个工作日，您处于静坐的时间大约为
平均每天___分钟 [填空题] *

28. 5c 过去 7 天，在每个休息日，您处于静坐的时间大约为
平均每天___分钟 [填空题] *

4. The Pittsburgh Sleep Quality Index (PSQI)

1. 在过去的一个月内您每天上床睡觉的时间是?(请按 24 小时制填写)___*:___	
2. 在过去的一个月内您每天上床睡觉的时间是?(请按 24 小时制填写)___:___*	
3. 在过去的一个月内您每晚通常要花费多长时间才能入睡?	1=≤15 分钟 2=16～30 分钟 3=31～60 分钟 4=＞60 分钟
4. 在过去一个月内您通常每天早上起床的时间是?（请按 24 小时制填写）___*:___	
5. 在过去一个月内您通常每天早上起床的时间是?（请按 24 小时制填写）___:___*	
6. 在过去一个月内您每晚通常实际的睡眠时间是多长？(不等于卧床时间)*hmin	
7. 在过去一个月内您每晚通常实际的睡眠时间是多长？(不等于卧床时间)h*min	
8. 过去一个月内您不能在 30 分钟内入睡?	1=无 2=＜1 次/周 3=1～2 次/周 4=≥3 次/周
9. 过去一个月内您在晚上睡觉过程中易醒或早醒?	1=无 2=＜1 次/周 3=1～2 次/周 4=≥3 次/周
10. 过去一个月内您晚上有没有出现过起床去上厕所?	1=无 2=＜1 次/周 3=1～2 次/周 4=≥3 次/周
11. 过去一个月内夜间睡眠过程中您有没有出现过呼吸不畅?	1=无 2=＜1 次/周 3=1～2 次/周 4=≥3 次/周
12. 过去一个月内夜间睡眠过程中您有没有出现过咳嗽或打鼾?	1=无 2=＜1 次/周 3=1～2 次/周 4=≥3 次/周
13. 过去一个月内夜间睡眠过程中您有没有感觉到寒冷?	1=无 2=＜1 次/周 3=1～2 次/周 4=≥3 次/周
14. 过去一个月内夜间睡眠过程中您有没有感觉到太热?	1=无 2=＜1 次/周 3=1～2 次/周 4=≥3 次/周
15. 过去一个月内夜间睡眠过程中您有没有做过噩梦?	1=无 2=＜1 次/周 3=1～2 次/周 4=≥3 次/周
16. 过去一个月内夜间睡眠过程中您有没有出现过疼痛不适?	1=无 2=＜1 次/周 3=1～2 次/周 4=≥3 次/周
17. 过去一个月内夜间睡眠过程中您是否还因其他原因影响到睡眠?	1=无 2=＜1 次/周 3=1～2 次/周 4=≥3 次/周
18. 请您评价一下您过去一个月总体的睡眠质量?	1=很好 2=较好 3=较差 4=很差
19. 过去一个月内您是否需要经常服用医生开具的药物或自行到药店购买的药物才可以入睡?	1=无 2=＜1 次/周 3=1～2 次/周 4=≥3 次/周
20. 过去一个月内您常感到困倦吗？	1=无 2=＜1 次/周 3=1～2 次/周 4=≥3 次/周
21. 过去一个月内您做事情的精力不足吗?	1=没有 2=偶尔有 3=有时有 4=经常有

5. The Negative Acts Questionnaire-Revised (NAQ-R)

	从来没有 (1)	偶尔(1 个月以上发生 1 次) (2)	每月都有(每月至少发生 1 次) (3)	每周都有(每周至少发生 1 次) (4)	每天都有(每天至少发生 1 次) (5)
1. 因同事对您隐瞒信息，影响您的工作表现					
2. 因工作的原因被羞辱或嘲笑					
3. 安排您去做低于能力水平的工作					
4. 剥夺您的主要工作职责，安排您去做琐碎的不愉快的工作					
5. 散布关于您的流言蜚语					
6. 您受到其他同事的忽视、排斥或孤立					
7. 您的个人情况如习惯、背景、态度或私生活等受到侮辱或攻击					
8. 同事发怒时对您叫嚷，拿您当出气筒					
9. 对您有如下胁迫行为：用手指您、侵犯您的个人空间、推拉拖拽、挡路等					
10. 同事暗示您应该调离现在的工作岗位					
11. 同事反复提及您的过错或过失					
12. 您受到同事的冷落或敌意					
13. 反复批评您的工作和所作出的努力					
14. 对您的观点和意见不予理睬					
15. 您受到与您关系不好同事的戏弄					
16. 安排您去做不合理的工作、不能实现的目标或不能按期完成的工作					
17. 您受到同事的指责					
18. 您的工作被过分监督					
19. 您应该享有的权利得不到保障（如：病假、法定休息、出差补助）					
20. 您成为同事取笑、讽刺的对象					
21. 您承担超负荷的工作量					
22. 您受到暴力威胁或身体伤害					

6. The Perceived Social Support Scale (PSSS)

	极不同意(1)	很不同意(2)	稍不同意(3)	中立 (4)	稍同意(5)	很同意(6)	极同意(6)
1. 在我遇到问题时有些人（领导、亲戚、同事）会出现在我的身旁							
2. 我能够与有些人（领导、亲戚、同事）共享快乐与忧伤							
3. 我的家庭能够切实具体地给我帮助							
4. 在需要时我能够从家庭获得感情上的帮助和支持							
5. 当我有困难时有些人（领导、亲戚、同事）是安慰我的真正源泉							
6. 我的朋友们能真正的帮助我							
7. 在发生困难时我可以依靠我的朋友们							
8. 我能与自己的家庭谈论我的难题							
9. 我的朋友们能与我分享快乐与忧伤							
10. 在我的生活中有些人（领导、亲戚、同事）关心着我的感情							
11. 我的家庭能心甘情愿协助我作出各种决定							
12. 我能与朋友们讨论自己的难题							

7. The Trait Coping Style Questionnaire (TCSQ)

	肯定不是 (1)	多数不是 (2)	一般都是 (3)	多数是 (4)	肯定是 (5)
1. 能尽快地将不愉快忘掉					
2. 易陷入对事件的回忆和幻想之中而不能摆脱					
3. 当作事情根本未发生过					
4. 易迁怒于别人而经常发脾气					
5. 通常向好的方面想、想开些					
6. 不愉快的事很容易引起情绪波动					
7. 喜欢将情绪压在心底里，不表现出来，但又忘不掉					
8. 通常与类似的人比较，就觉得算不了什么					
9. 能较快将消极因素化为积极因素，例如参加活动					
10. 遇烦恼的事很容易想悄悄地哭一场					
11. 旁人很容易使你重新高兴起来					
12. 如果与人发生冲突，宁可长期不理对方					
13. 对重大困难往往举棋不定，想不出办法					
14. 对困难和痛苦能很快适应					
15. 相信困难和挫折可以锻炼人					
16. 在很长的时间里回忆所遇到的不愉快的事					
17. 遇到难题往往责怪自己无能而怨自己					
18. 认为天底下没有什么大不了的事					
19. 遇苦恼事喜欢一人独处					
20. 通常以幽默的方式化解尴尬局面					

8. Perceived Stress Scale (PSS)

	从不 (0)	偶尔 (1)	有时 (2)	经常 (3)	总是 (4)
1. 在过去一个月内，您是否经常会因为一些突发的事情，而让心情变得不好？					
2. 在过去一个月内，您是否经常会觉得您没有办法控制生活里一些重要的事情？					
3. 在过去一个月内，您是否经常会觉得紧张而且有压力？					
4. 在过去一个月内，您对自己处理个人问题的能力，是否经常会觉得有信心？					
5. 在过去一个月内，您是否经常会觉得一切事情都很顺心如意？					
6. 在过去一个月内，您是否经常会觉得无法应付您必须要做的事？					
7. 在过去一个月内，对于生活中一些容易惹人生气的小事情，您是否经常能控制得宜？					
8. 在过去一个月内，您是否经常会觉得事情都在您可以控制的范围内？					
9. 在过去一个月内，您是否经常会因为一些您无法控制的事情而让你感到生气？					
10. 在过去一个月内，您是否经常觉得困难太多让您无法解决？					

9. The Generalized Anxiety Disorder 7-Item Scale (GAD-7)

	完全不会（0）	有几天（1）	一半以上天数（2）	几乎每天（3）
1. 在过去的两周内，以下情况烦扰您有多频繁?_感觉紧张，焦虑或急切				
2. 在过去的两周内，以下情况烦扰您有多频繁?_不能够停止或控制担忧				
3. 在过去的两周内，以下情况烦扰您有多频繁?_对各种各样的事情担忧过多				
4. 在过去的两周内，以下情况烦扰您有多频繁?_很难放松下来				
5. 在过去的两周内，以下情况烦扰您有多频繁?_由于不安而无法静坐				
6. 在过去的两周内，以下情况烦扰您有多频繁?_变得容易烦恼或急躁				
7. 在过去的两周内，以下情况烦扰您有多频繁?_感到似乎有可怕的事发生而害怕				

10. The 9-item Patient Health Questionnaire (PHQ-9)

	完全不会（0）	有几天（1）	一半以上天数（2）	几乎每天（3）
1. 在过去的两周内，以下情况烦扰您有多频繁?_做什么事都没兴趣，没意思				
2. 在过去的两周内，以下情况烦扰您有多频繁?_感到心情低落，抑郁，没希望				
3. 在过去的两周内，以下情况烦扰您有多频繁?_入睡困难，总是醒着或睡得太多、嗜睡				
4. 在过去的两周内，以下情况烦扰您有多频繁?_常感到很疲倦，没劲				
5. 在过去的两周内，以下情况烦扰您有多频繁?_口味不好，或吃的太多				
6. 在过去的两周内，以下情况烦扰您有多频繁?_自己对自己不满，觉得自己是个失败者，或让家人丢脸了				
7. 在过去的两周内，以下情况烦扰您有多频繁?_无法集中精力，即便是读报纸或看电视时，记忆力下降				
8. 在过去的两周内，以下情况烦扰您有多频繁?_行动或说话缓慢到引起人们的注意，或刚好相反，坐立不安，烦躁易怒，到处走动				
9. 在过去的两周内，以下情况烦扰您有多频繁?_有不如一死了之的念头，或想怎样伤害自己一下				
